# Spatiotemporal inventory of PFOS and PFOA in China’s air, water and soil (2016–2021): Sharp declines yet persistent hotspots

**DOI:** 10.1016/j.ese.2026.100785

**Published:** 2026-10-29

**Authors:** Shuyu Ouyang, Min Wu, Ke Jiang, Rongcan Chen, Ying Zhu, Huizhong Shen, Hefa Cheng, Bin Wang, Guofeng Shen, Shu Tao

**Affiliations:** a College of Urban and Environmental Sciences, Peking University, Beijing, 100871, China; b State Environmental Protection Key Laboratory of Environmental Health Impact Assessment of Emerging Contaminants, Shanghai Jiao Tong University, Shanghai, 200240, China; c School of Environmental Science and Engineering, Shanghai Jiao Tong University, Shanghai, 200240, China; d School of Environmental Science and Engineering, Southern University of Science and Technology, Shenzhen, 518055, China; e Guangdong Provincial Observation and Research Station for Coastal Atmosphere and Climate of the Greater Bay Area, Shenzhen, 518055, China; f Institute of Reproductive and Child Health, Peking University, Beijing, 100191, China; g Institute of Carbon Neutrality, Peking University, Beijing, 100871, China; h Center for Environment and Health, Peking University, Beijing, 100871, China

**Keywords:** PFOA, PFOS, Emission inventory, High resolution, Multimedia hotspots

## Abstract

Perfluorooctane sulfonate (PFOS) and perfluorooctanoic acid (PFOA) are persistent organic pollutants regulated under the Stockholm Convention, yet their national emission trajectories and spatiotemporal dynamics in China remain poorly constrained. Here we develop a bottom-up, consecutive 0.1-degree inventory across air, water, and soil from 2016 to 2021, showing that China’s PFOS emissions declined by 89%, from 49 (40–55) to 5.2 (4.3–5.9) t yr^−1^, while PFOA emissions fell by over 99%, from 75 (62–90) to 0.31 (0.25–0.37) t yr^−1^. These sharp declines coincided with regulatory restrictions and technological transitions across fluoropolymer manufacturing, metal plating, aqueous film-forming foams, and sulfluramid pesticides. The decline also reshaped the spatial structure of releases, shifting the emission profile from geographically dispersed pesticide and industrial use toward localized clusters at industrial and firefighting sites. Water remained the principal receiving medium (60% for PFOS and 71% for PFOA), and residual hotspots overlapped with dense populations in eastern and southeastern industrial zones. This inventory provides a quantitative basis for multimedia fate modeling, hotspot mitigation, and policy evaluation.

## Highlights

• A consecutive 0.1-degree inventory tracks PFOS and PFOA in air, water, and soil.
• National PFOS and PFOA emissions fell by 89% and over 99% from 2016 to 2021.
• Sources shifted from production and pesticides to firefighting and plating.
• Grid emission densities contracted sharply yet eastern clusters remained.
• Persistent emission hotspots overlap dense populations in major river deltas.

## Graphical abstract

**Figure:**
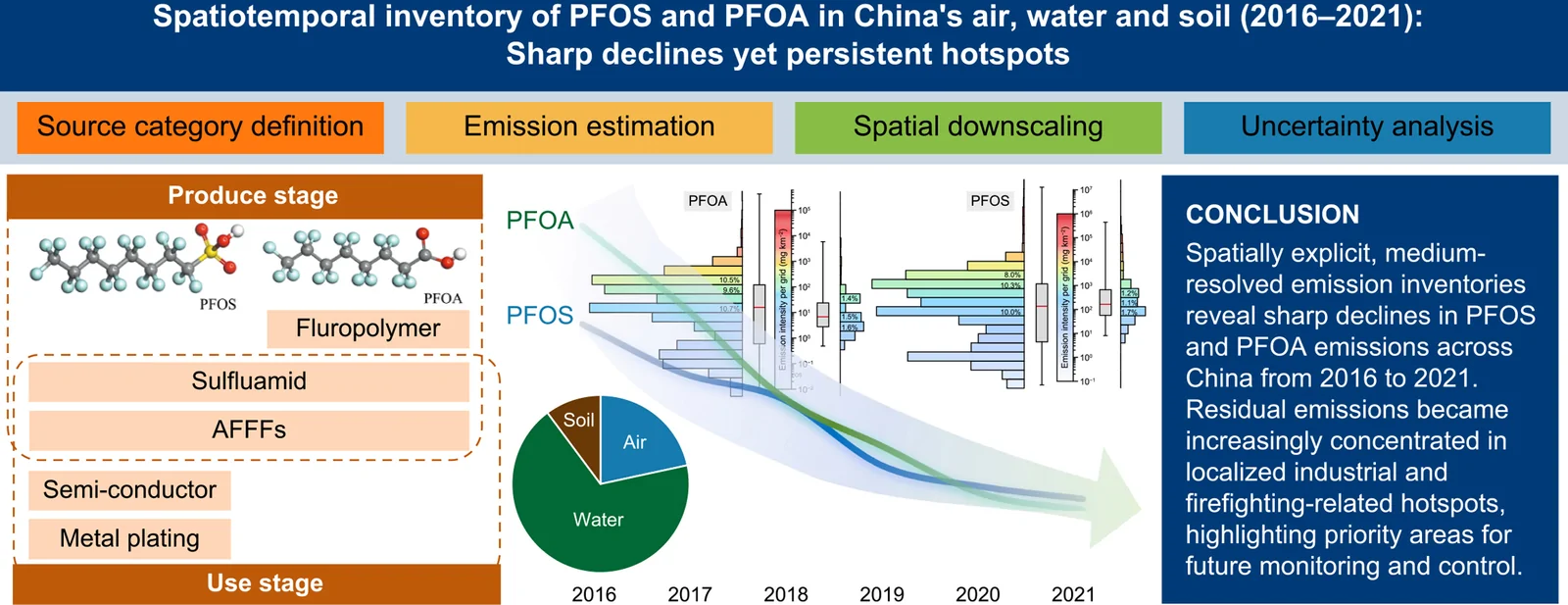

## 1 Introduction

Per- and polyfluoroalkyl substances (PFAS) are a class of anthropogenic organofluoride compounds that have been extensively used in industrial applications and consumer products since the mid-twentieth century [1]. PFAS are ubiquitous in water, atmospheric aerosols, soils, and biological tissues, underscoring their widespread dispersion and cross-media transport [1], [2]. Their persistence in environmental media and biota raises substantial concerns regarding ecosystem and human health [3]. Among PFAS, perfluorooctane sulfonate (PFOS) and perfluorooctanoic acid (PFOA) are well-studied legacy compounds that are widely detected and frequently reported at comparatively high concentrations. They are therefore considered priority contaminants of global concern [4], [5], [6], [7], [8].

Dietary intake and ingestion of contaminated drinking water are major human exposure pathways for PFOS and PFOA [9], which can accumulate in serum and organs such as the liver and bone [10]. Several epidemiological and toxicological investigations have demonstrated associations between PFOS and PFOA exposure and a range of adverse health outcomes, including immunotoxicity, endocrine disruption, developmental impairments, hepatic and renal dysfunction, and elevated risks of certain cancers [11], [12], [13], [14]. For example, a meta-analysis reported a positive association between circulating PFOA and the risk of type 2 diabetes [15]. Although uncertainties remain regarding precise causal mechanisms and dose-response relationships, a substantial body of evidence indicates that PFOS and PFOA may pose significant risks to human health. PFOS and PFOA are listed under the Stockholm Convention and are subject to international restrictions, although the scope and timing of implementation vary by jurisdiction [16], [17]. In China, the Ministry of Ecology and Environment has designated PFOS and PFOA as priority control chemicals and implemented a suite of regulatory measures, including *the China PFOS Priority Sectors Reduction and Phase-Out Project* and bans on the production, distribution, and use of PFOS and its precursors, except for specific authorized applications [18], [19], [20]. Despite these measures, their persistence and widespread historical use have created extensive environmental reservoirs, posing continuing challenges for remediation and risk management [2], [3].

Although PFOS production and use have been restricted or phased out in many jurisdictions, substantial quantities continue to be produced or used in regions where cost-effective alternatives and effective enforcement remain limited [21]. Robust emission inventories are therefore needed to quantify releases, identify major sources and spatial hotspots, evaluate control measures, and support environmental and human health risk assessments. To date, few global or regional PFAS emission inventories have been developed. Existing studies generally identify industrial activities as the dominant quantifiable sources of PFAS releases, with smaller estimated contributions from municipal wastewater and solid-waste streams [21], [22], [23], [24]. At the global scale, Simon et al. [25] compiled a 0.5° × 0.5° PFAS emission inventory for 1950–2020 that included releases from airport firefighting, fluoropolymer production, and diffuse product-related sources. The dataset provided a long-term global framework, but its sectoral coverage was limited, and it considered only emissions to air and water. Moreover, the increasing trend reported for China differs from the declining patterns observed in several recent Chinese monitoring studies [26], [27], [28]. At the national or regional scale, Xie et al. [21] developed a framework across detailed industrial categories, including PFOS production, textile treatment, metal plating, firefighting foams, and semiconductors, and estimated total PFOS-equivalent releases from major industrial sources in China at approximately 70 tons in 2010. Liu et al. [23] estimated total PFOS and PFOA releases in central and eastern China at approximately 70 and 40 t yr^−1^, respectively. However, the study lacked detailed sectoral disaggregation, and industrial PFOA releases were estimated primarily from wastewater-related pathways to water and soil. Du et al. [29] further estimated industrial atmospheric emissions of PFOA and its salts in China at approximately 38.2 t in 2019. The primary sources were fluoropolymer production, in which PFOA is used as a processing aid, and PFOA released as a reaction by-product during perfluorooctane sulfonyl fluoride (PFOSF)-derivative and fluorotelomer synthesis. Collectively, these studies provide important benchmarks, but most cover a single year or selected regions and do not resolve consecutive annual trends, medium-specific releases, or national spatial heterogeneity.

The absence of a nationally consistent, high-resolution, consecutive-year multimedia inventory has limited analyses of PFOS and PFOA emission dynamics and the identification of priority control areas. In this study, we quantified PFOS and PFOA emissions in China from 2016 to 2021 using industrial production and use data, literature-derived emission factors, and official statistics. Relative to previous inventories, our framework provides consecutive annual coverage at a nationwide spatial resolution of 0.1° × 0.1°, detailed source sectors, and explicit allocation of emissions to air, water, and soil. It also applies source-specific downscaling proxies, including land-use data for sulfluramid applications and fire statistics combined with relevant facility locations for aqueous film-forming foam (AFFF) use. On this basis, we examined temporal changes in total and medium-specific emissions, sectoral contributions, and grid-level spatial heterogeneity. The resulting inventory identifies priority source regions and provides spatially and temporally resolved source terms for future multimedia fate modeling, exposure assessment, and evaluation of emission-control policies.

## 2 Materials and methods

### 2.1 Framework and data sources

To quantify PFOS and PFOA emissions across the Chinese mainland, we constructed a bottom-up multimedia emission inventory for 2016–2021 at a spatial resolution of 0.1° × 0.1°. The inventory covers the major quantifiable industrial source categories identified in previous studies and focuses on two types of environmental releases: unintentional emissions from manufacturing facilities producing PFAS-containing substances and process-related emissions from manufacturing products in which PFAS serve as functional components or processing aids.

For PFOS, we included five source categories: PFOS manufacturing; the manufacture and use of AFFFs; the manufacture and use of sulfluramid (N-ethyl perfluorooctane sulfonamide, Et-FOSA) in the pesticide sector; the use of PFOS-based mist suppressants in metal plating; and photoresist use in the semiconductor sector. For PFOA, we included four categories: PFOA manufacturing; fluoropolymer production using PFOA as a processing aid; the manufacture and use of PFOA-based AFFFs; and sulfluramid use in the pesticide sector. Industrial sources for which sufficient activity data were unavailable, as well as residential sources, were excluded from the inventory. Detailed descriptions of all source categories are provided in Supplementary Text S1. The 0.1° × 0.1° resolution balances representing localized hotspots with the precision of area-source proxies, avoids the artificial precision of finer grids, and remains suitable for subsequent multimedia modeling.

We integrated source-specific activity data and literature-derived emission factors to estimate annual PFOS and PFOA emissions. We then allocated the estimated emissions among air, water, and soil; spatially disaggregated them to 0.1° × 0.1° grid cells using facility locations and source-specific spatial proxies; and subjected them to Monte Carlo uncertainty and sensitivity analyses (Fig. 1).

**Fig. 1:**
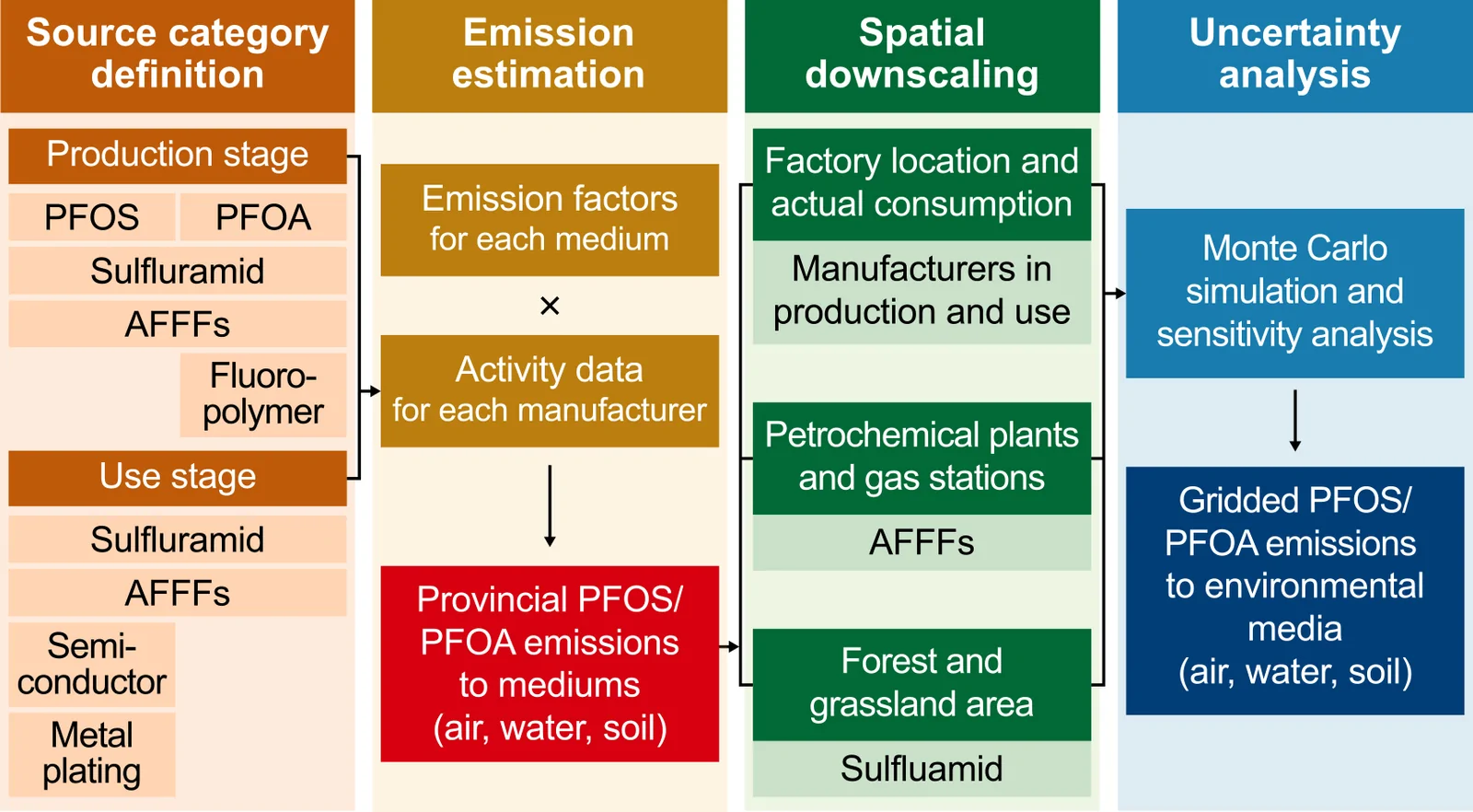
**Workflow for developing spatially explicit PFOS and PFOA emission inventories**. The workflow integrates source categorization, emission estimation, spatial downscaling, and uncertainty analysis to generate emission inventories resolved by environmental medium. PFOS, perfluorooctane sulfonate; PFOA, perfluorooctanoic acid; AFFFs, aqueous film-forming foams.

Within this framework, we converted releases of PFOS-related precursor chemicals to PFOS-equivalent emissions using reported transformation factors, and derived PFOA emissions attributable to the Et-FOSA pathway from reported PFOA content values and transformation parameters.

For most source categories, annual emissions were estimated using equation (1):

(1) $$E_{s,y,m}=A_{s,y}\times F_{s,m}$$

where *E*_*s,y,m*_ denotes annual emissions (kg yr^−1^) to environmental medium *m* from source category *s* in year *y*, *A*_*s,y*_ denotes the corresponding annual activity level for source category *s* in year *y,* expressed in source-specific units (t yr^−1^ or kg yr^−1^), and *F*_*s,m*_ denotes the source-specific factor or allocation coefficient used to estimate emissions to medium *m,* expressed in units consistent with the activity data and emission output (e.g., kg t^−1^ or dimensionless). All quantities were converted to consistent mass units when necessary.

For pathways involving both production and use stages, such as aqueous film-forming foams and sulfluramid-related pathways, stage-specific emissions were estimated separately and subsequently summed. Sulfluramid-related emissions were estimated using equations (2), (3), respectively:

(2) $$E_{\text{PFOS},\text{use},\text{sul},y,m}=Q_{y}\times C_{\text{sul}}\times R_{\text{trans},\text{PFOS}}\times F_{\text{use},\text{sul},m}$$

(3) $$E_{\text{PFOA},\text{use},\text{sul},y,m}=Q_{y}\times C_{\text{sul}}\times \left(R_{\text{content},\text{PFOA}}+R_{\text{trans},\text{PFOA}}\right)\times F_{\text{use},\text{sul},m}$$

where *E*_PFOS,use,sul*,y,m*_ and *E*_PFOA,use,sul*,y,m*_ denote annual PFOS and PFOA emissions from sulfluramid use to medium *m* in year *y* (kg yr^−1^); *Q*_*y*_ represents annual sulfluramid production (kg yr^−1^); *C*_sul_ is the mass fraction of Et-FOSA in commercial sulfluramid products (dimensionless); *R*_trans, PFOS_ and *R*_trans, PFOA_ denotes the mass of PFOS and PFOA formed per unit mass of initial Et-FOSA (dimensionless); *R*_content, PFOA_ is the initial PFOA mass fraction in the perfluorinated feedstock used to produce Et-FOSA (dimensionless). The Et-FOSA mass calculated from product production and purity was used as an approximate mass basis for these PFOA-related fractions. *F*_use,sul,*m*_ is the medium-specific emission allocation coefficient (dimensionless).

Supplementary Text S1 provides sector-specific definitions of activity data, detailed data sources, and spatial allocation procedures; Supplementary Table S1 summarizes the corresponding emission factors, conversion parameters, uncertainty ranges, and literature sources. We compiled activity data for the Chinese mainland from industry research reports, official statistical yearbooks, enterprise registry records, and facility-level datasets. Emission factors were obtained from previous PFAS inventories for China and source-specific studies. Contemporary, China-specific, process-level emission factors remain unavailable for several source categories. We therefore retained the best available literature-derived factors and propagated their uncertainties through Monte Carlo simulation (Section 2.2). When only national totals were available, emissions were allocated among provinces using source-specific normalized proxies and then downscaled to grid cells using facility coordinates or source-relevant spatial surrogates (Supplementary Fig. S1). Where plant-specific abatement information was unavailable, no additional removal efficiency was applied. The inventory is observational. Regulatory and technological developments were used to interpret temporal patterns, but their individual causal effects were not estimated unless explicitly represented in the activity data or emission factors.

To identify priority areas where elevated emissions spatially coincide with large populations, a population-weighted emission index was calculated using equations (4), (5):

(4) $$w_{i}=\frac{P_{i}}{\sum_{j=1}^{n}P_{j}}$$

(5) $$I_{i}=D_{i}\times w_{i}$$

where *P*_*i*_ and *P*_*j*_ denote the population counts (persons) in grid cell *i* and *j*, respectively; *n* is the total number of grid cells included in the normalization (dimensionless); and *w*_*i*_ is the corresponding population proportion (dimensionless). *D*_*i*_ is the annual PFOS or PFOA emission density in grid cell *i* (kg km^−2^ yr^−1^), and *I*_*i*_ is the population-weighted emission density (kg km^−2^ yr^−1^). Population counts from the *China Population Spatial Distribution Kilometer Grid Dataset* [30] were aggregated to the 0.1° × 0.1° inventory grid before normalization. The resulting *I*_*i*_ represents a screening-level indicator of spatial overlap between population and emissions rather than a measure of actual exposure or health risk.

### 2.2 Uncertainty analysis

We quantified uncertainty in the emission estimates using Monte Carlo simulation with 10,000 iterations. Input parameters were assigned probability density functions based on data availability. Parameters without explicitly reported ranges were modeled using lognormal distributions with a conservative coefficient of variation of 0.1, whereas parameters with reported ranges were assigned uniform distributions. To ensure mass conservation, medium-specific allocation fractions were normalized to sum to one. Output uncertainty was summarized using 95% confidence intervals, calculated from the 2.5th and 97.5th percentiles of the Monte Carlo results. We performed a sensitivity analysis using squared Spearman rank-correlation coefficients to quantify each parameter’s contribution to total inventory variance.

Because grid-cell emissions were strongly right-skewed, spatial heterogeneity was summarized using the relative variability index (RVI), defined as the interquartile range (IQR) divided by the median. Annual grid-level RVI statistics are provided in Supplementary Table S2.

## 3 Results

The inventory was organized according to emission stage, source sector, and receiving medium (water, air, and soil; Fig. 2).

**Fig. 2:**
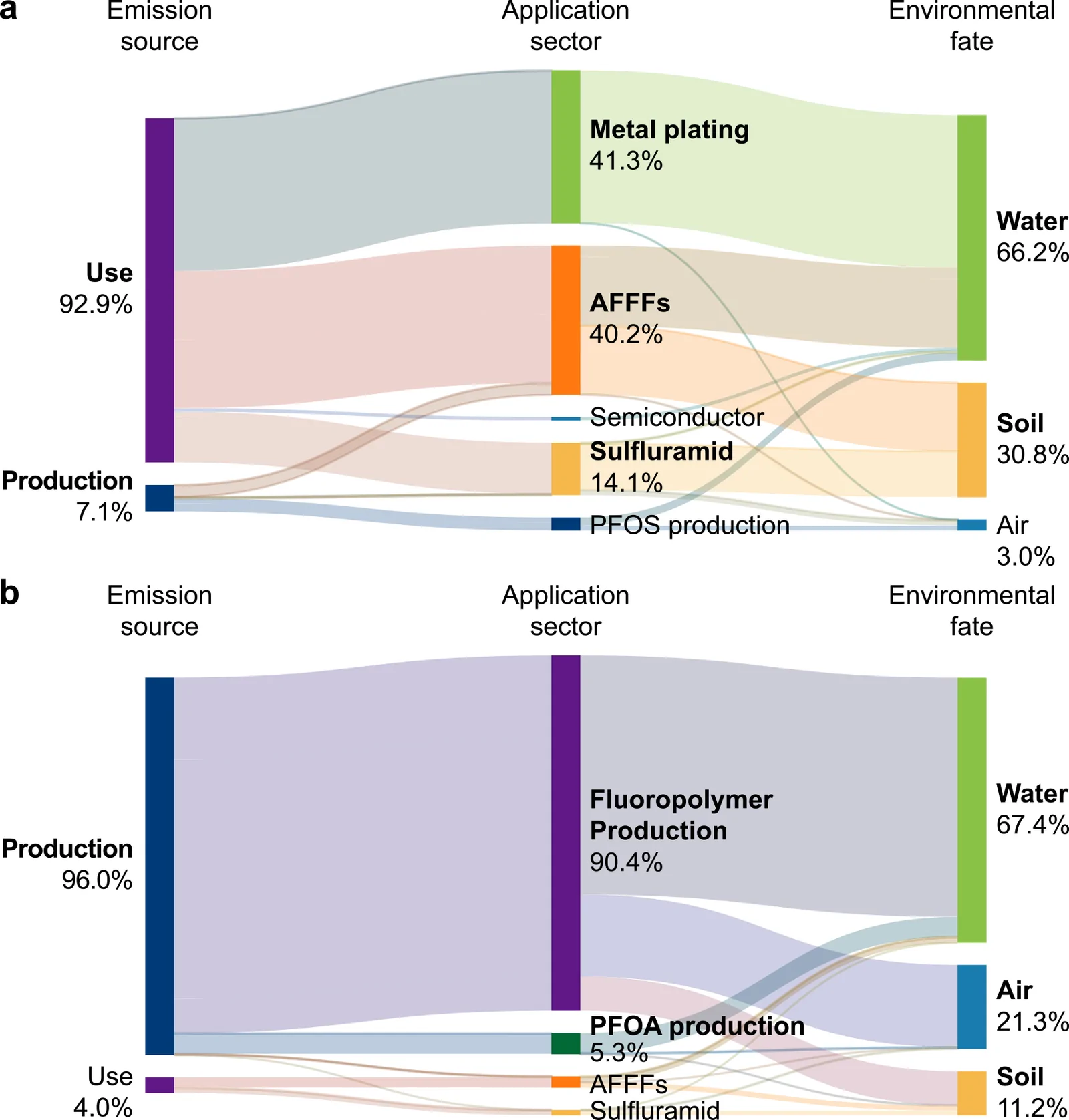
**Composition of PFOS and PFOA emission inventories by source stage, application sector, and environmental medium in 2016**. Sankey diagrams show the proportional contributions of emission stages (production and use), application sectors, and receiving environmental media (water, air, and soil) for PFOS (**a**) and PFOA (**b**). Percentages represent the contributions of individual categories to the corresponding PFOS/PFOA national total emissions in 2016. Boldface highlights categories with displayed percentages, which are shown for all emission stages and environmental media and selected major source sectors to improve readability. AFFFs, aqueous film-forming foams; PFOS, perfluorooctane sulfonate; PFOA, perfluorooctanoic acid.

### 3.1 Changes in total emissions and source shares

Both PFOS and PFOA emissions declined substantially during the study period, with different sectors contributing to these reductions (Fig. 3). National PFOS emissions decreased sharply from 49 t yr^−1^ (95% CI, 40–55 t yr^−1^) in 2016 to 5.2 t yr^−1^ (4.3–5.9 t yr^−1^) in 2021, representing an overall reduction of approximately 89%. In 2016, PFOS emissions were dominated by use-related releases (93%), whereas production-related releases accounted for only 7%. At the sectoral level, metal plating and AFFFs were the two largest contributors in 2016, accounting for 41% and 40% of national PFOS emissions, respectively. By 2021, the source composition had changed substantially, with AFFFs becoming the dominant remaining source and accounting for 62% of total PFOS emissions. The metal plating and AFFF sectors drove the largest numerical declines in PFOS emissions, accounting for 44% and 37% of the total reduction, respectively. The timing of these declines coincided with the policy and technological developments summarized in Supplementary Table S3 [17], [18], [19], [31], [32], [33], [34], [35], [36].

**Fig. 3:**
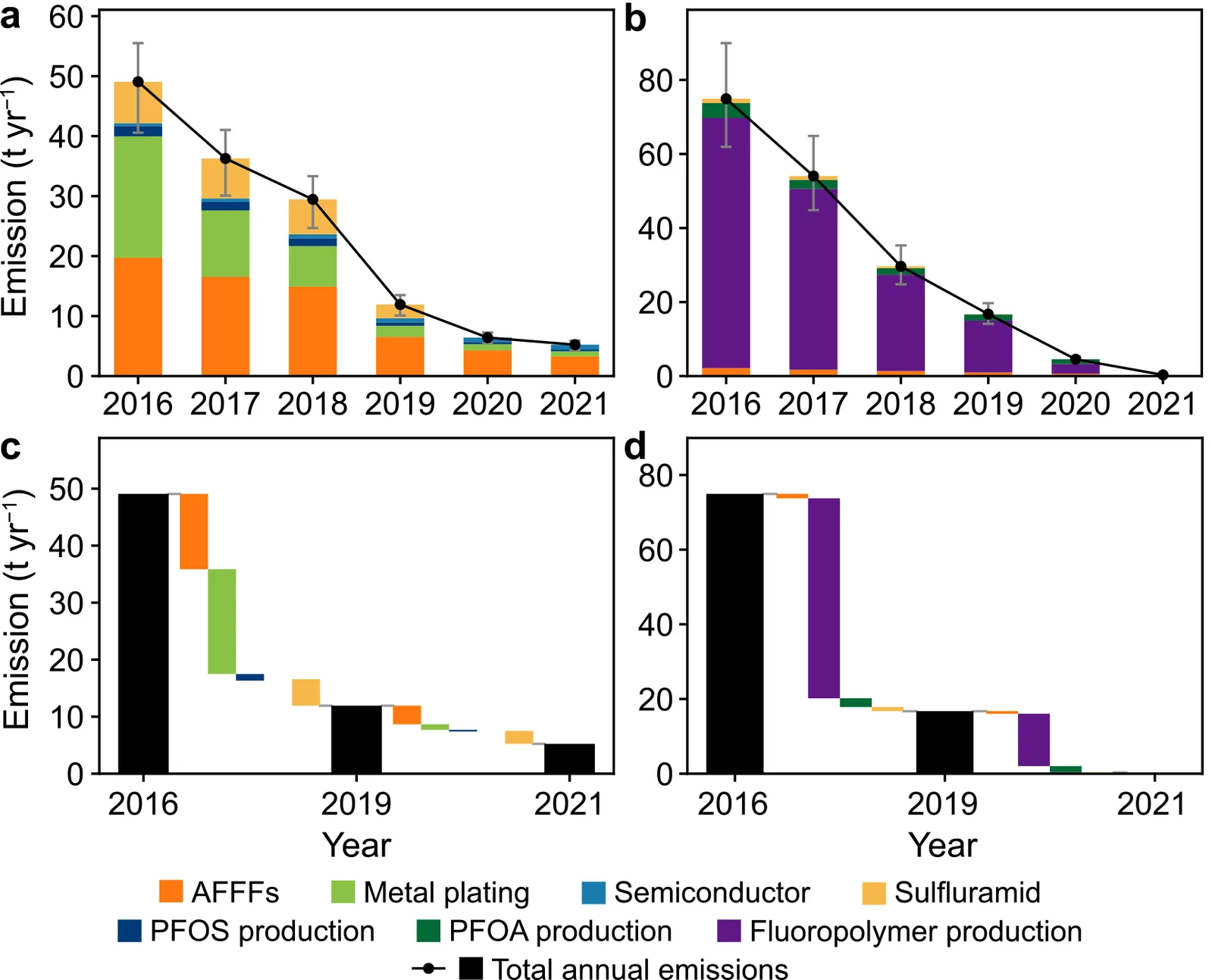
**Temporal trends in annual national emissions and sectoral contributions to emission reductions for PFOS and PFOA, 2016–2021. a**–**b**, Total annual emissions disaggregated by source sector for PFOS (**a**) and PFOA (**b**). Error bars represent the 95% uncertainty range. **c**–**d**, Waterfall decomposition of cumulative emission reductions attributed to each source sector between 2016 and 2021 for PFOS (**c**) and PFOA (**d**). PFOS, perfluorooctane sulfonate; PFOA, perfluorooctanoic acid; AFFFs, aqueous film-forming foams.

PFOA emissions declined even more sharply, from 75 t yr^−1^ (62–90 t yr^−1^) in 2016 to 0.31 t yr^−1^ (0.25–0.37 t yr^−1^) in 2021, corresponding to an overall reduction of more than 99%. In 2016, PFOA emissions were overwhelmingly dominated by production-related releases (96%), whereas use-related releases accounted for only 4%. Fluoropolymer production was the major source of PFOA emissions in 2016, contributing 90% of the national total. By 2021, however, the source profile had shifted markedly, with AFFFs accounting for nearly all residual PFOA emissions. Fluoropolymer production accounted for the largest numerical contribution to the overall decline. The timing of this decline coincided with the reported adoption of alternative emulsifiers, PFOA recovery and recycling, and improvements in waste management [37], [38]. Key policy milestones and their temporal correspondence with sectoral emission trends are summarized in Supplementary Table S3.

### 3.2 Spatially explicit distribution and emission reductions

PFOS and PFOA showed similar broad-scale spatial patterns, although their point-source hotspots differed in location and source composition. Emission densities were generally higher in eastern and southeastern China than in western China. Four major high-emission regions were evident in 2016, including the Yellow River Basin, the Bohai Rim, the Yangtze River Delta (YRD), and the Pearl River Delta (PRD) (Supplementary Fig. S2). These regions contain intensive industrial activity and multiple PFOS- and PFOA-related emission sources.

In 2016, PFOS emissions were concentrated in the Yellow River Basin, the Bohai Rim, the middle and lower YRD, and the PRD (Supplementary Fig. S2). The maximum grid-level emission density exceeded 50 kg km^−2^ yr^−1^. Several high-intensity grid cells were associated with metal plating facilities. PFOS emissions declined substantially from 2016 to 2021. By 2021, emission densities were below 1.0 kg km^−2^ yr^−1^ across most emitting grid cells, although residual clusters remained in the Bohai Rim, the YRD, and the PRD. The decline reduced both the intensity and spatial extent of existing emission clusters but did not eliminate their regional concentration.

PFOA emissions showed a similar east- and southeast-oriented pattern in 2016 (Supplementary Fig. S2). Major clusters occurred in the abovementioned four regions. Several elevated grid cells were also located near PFOA and fluoropolymer production facilities. The maximum emission density approached 200 kg km^−2^ yr^−1^ in 2016 but decreased to approximately 0.15 kg km^−2^ yr^−1^ by 2021. Most former PFOA clusters disappeared, leaving only a few isolated emitting grid cells. This spatial contraction was stronger than that observed for PFOS.

The grid-level distributions of both compounds were strongly right-skewed (Fig. 4). Most grid cells with nonzero emissions had low emission densities, whereas a small number of high-intensity grid cells formed the upper tails of the distributions. For PFOS, many grid cells with nonzero emissions had densities within the range of 0.01–1 kg km^−2^ yr^−1^ in 2016. The upper tail contracted markedly by 2021, with most values falling below 1 kg km^−2^ yr^−1^. PFOA exhibited a more pronounced downward shift, with nearly all nonzero PFOA emission densities falling below 0.1 kg km^−2^ yr^−1^ in 2021. The histograms show a redistribution of grid cells with nonzero emissions toward lower emission-density intervals, while the boxplots show decreases in the median, IQR, and overall range.

**Fig. 4:**
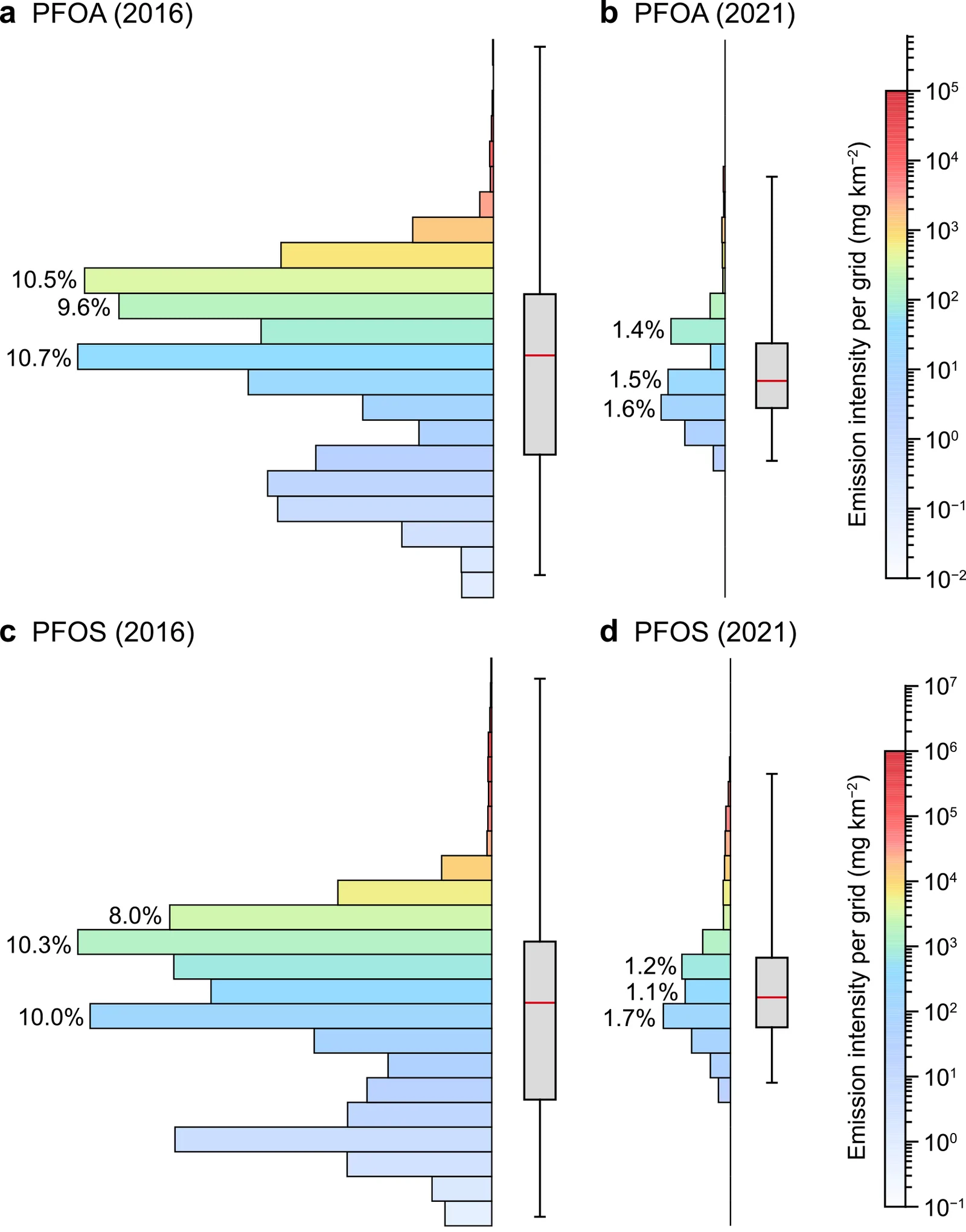
**Grid-level emission-intensity distributions of PFOS and PFOA changed between 2016 and 2021. a**–**b**, Distributions of grid-level PFOA emission intensities in 2016 (**a**) and 2021 (**b**). **c**–**d**, Distributions of grid-level PFOS emission intensities in 2016 (**c**) and 2021 (**d**). Histograms show the percentage distributions of non-zero-emission grid cells across emission-intensity intervals, with successive bin boundaries differing by a factor of two. Horizontal bar lengths represent grid-cell counts, while percentage annotations indicate the proportions of all grid cells falling within the three most populated intervals. Adjacent box plots summarize the distributions of grid-level non-zero-emission intensities, with red lines indicating medians, grey boxes representing the interquartile ranges (25–75th percentiles), and whiskers indicating the minimum and maximum plotted values after extreme-value clipping. The color scales indicate emission intensity per grid (mg km^−2^). PFOS, perfluorooctane sulfonate; PFOA, perfluorooctanoic acid.

Spatial patterns of absolute and relative emission reductions are presented in Supplementary Fig. S3. Point sources generally showed larger reductions than area sources in both absolute magnitude and relative percentage. By 2017, emissions from many point sources had declined by more than 50% relative to 2016, whereas reductions from most area sources were approximately 20–40%. The largest absolute reductions occurred mainly in the PRD and YRD and were more pronounced for PFOS than for PFOA, indicating greater absolute reductions at major PFOS point sources. The largest relative reductions in 2017 occurred around point sources in Tianjin, Hebei, Shandong, northern Zhejiang, Sichuan, and the northeastern provinces of Liaoning, Jilin, and Heilongjiang. PFOS and PFOA exhibited similar patterns of relative reduction. Hotspots of relative reduction were more spatially clustered than those of absolute reduction. This pattern may reflect concurrent declines among nearby point sources, although the available data do not resolve the underlying facility-level mechanisms. By 2021, PFOS and PFOA emissions had declined by more than 80% relative to 2016 across most of the Chinese mainland.

Grid-level emissions exhibited pronounced spatial heterogeneity throughout the study period. To quantify relative spatial heterogeneity, the RVI was calculated as the ratio of the IQR to the median grid-level emissions for each year (Supplementary Table S2). For both PFOA and PFOS, the RVI remained high and relatively stable from 2016 to 2019, declined markedly in 2020, and partially rebounded in 2021. Thus, spatial heterogeneity did not decrease monotonically. Instead, relative variability declined in 2020–2021 but remained substantial. The partial rebound in 2021 indicates persistent localized differences among emitting grid cells despite the overall national decline. This heterogeneity may reflect regional differences in industrial structure and the spatial concentration of source-specific activities. Fluorochemical manufacturing, metal plating, petrochemical production and storage, and firefighting-related activities are concentrated mainly in industrialized eastern and coastal regions. Differences in production technologies, closed-loop recovery systems, wastewater pretreatment, and treatment efficiency may further influence release fractions from similar activities. The timing and extent of industrial retrofits, chemical substitution, facility closures, and local implementation of regulatory requirements may also contribute to the remaining variability. However, facility- and region-specific data on wastewater-treatment performance and regulatory enforcement were unavailable and were not parameterized separately. These factors are therefore considered plausible modifiers rather than quantitatively attributed drivers.

### 3.3 Medium-specific emission distributions and temporal trends

Emissions were further allocated among water, air, and soil to characterize medium-specific release pathways and temporal trends (Supplementary Fig. S4). Approximately 60% (52–68%) of PFOS emissions were released to water, 36% (29–44%) to soil, and 3% to air. Emissions to all three media declined over similar periods. Atmospheric PFOS emissions accounted for only a small fraction of total emissions and decreased from 1.5 t in 2016 to 0.12 t in 2021. PFOS manufacturing and sulfluramid use in pesticides were the principal sources of atmospheric PFOS emissions. Sulfluramid-related emissions declined sharply after 2019 and were set to zero in the inventory beginning in 2020, following the prohibition on sulfluramid use under Announcement No. 148 [32]. Aqueous PFOS emissions declined from 32 t in 2016 to 3.6 t in 2021. The largest decrease occurred after 2019 and coincided with restrictions limiting PFOS use to closed-loop electroplating systems [18]. AFFFs and metal plating were the main contributors to aqueous PFOS emissions before 2019; AFFFs surpassed metal plating as the primary source thereafter. The decline in PFOS soil emissions was largely attributable to the cessation of sulfluramid-related releases following Announcement No. 148 [32], consistent with the concurrent decline in atmospheric PFOS emissions.

PFOA emissions were released primarily to water (71%), followed by air (17%) and soil (12%). Atmospheric PFOA emissions declined substantially and shifted from fluoropolymer production-related releases to AFFF-derived emissions by the end of the study period. By 2021, estimated PFOA emissions from non-AFFF sources had declined to near zero. This temporal pattern coincided with documented industrial substitution, improvements in recovery and recycling, and the inclusion of PFOA and related compounds in the *2020 List of Priority Controlled Chemicals* (Supplementary Table S3) [19]. For aqueous emissions, fluoropolymer production was initially the dominant source, but only AFFF-related releases remained by 2021. PFOA emissions to soil remained relatively low throughout the study period, with the source shifting from fluoropolymer production (65% in 2016) to firefighting foam use, while emissions from other sources declined to near zero by 2021.

Although total PFOS and PFOA emissions showed broadly similar spatial patterns, their medium-specific distributions differed because provincial PFAS-related activities varied (Supplementary Fig. S5). Atmospheric PFOS emissions concentrated in central, southern, and northeastern regions, whereas PFOA emissions concentrated in central and eastern provinces. The overall spatial structure of atmospheric emissions remained stable, but total quantities declined sharply over time. From 2016 to 2019, point-source emissions from PFOS and PFOA manufacturing plants declined markedly, coinciding with strengthened national restrictions. By 2020–2021, estimated atmospheric PFOS and PFOA emissions had nearly ceased nationwide, with nonzero emissions remaining in only a small number of grid cells. For soil emissions, PFOS hotspots were largely confined to eastern regions, whereas PFOA spatial patterns shifted markedly over time. From 2016 to 2019, most high-emission grid cells were located east of the Heihe–Tengchong Line (also known as the Hu Huanyong line), consistent with population and industrial density. After sulfluramid use was prohibited, PFOA soil emissions from this precursor pathway became concentrated in south-central provinces such as Jiangxi and Guangxi. For aqueous emissions, PFOS and PFOA exhibited substantial regional overlap, with elevated levels in southern and central areas. PFOS also showed a higher density of metal-plating point sources in eastern regions that remained active through 2021, consistent with continued PFOS use in closed-loop electroplating operations permitted under national regulations [18]. These patterns indicate a progressive shift toward localized, industry-driven, AFFF-related PFOS and PFOA releases in recent years.

We conducted an exploratory correlation analysis to examine bivariate relationships between city-level PFOS and PFOA emissions and economic activity (Supplementary Fig. S6). PFOA emissions showed a moderate positive correlation with agricultural gross domestic product (GDP; Pearson’s *r* = 0.45), whereas PFOS emissions were moderately correlated with total GDP and industrial GDP (Pearson’s *r* = 0.47 and 0.43, respectively). Cities with greater agricultural activity tended to have higher estimated PFOA emissions, whereas estimated PFOS emissions tended to increase with total and industrial GDP. Because the analysis did not control for population density, industrial composition, source-specific activity, treatment efficiency, or regulatory conditions, these correlations should be interpreted only as descriptive covariation and not as evidence that GDP independently determines emissions or explains the underlying mechanisms.

## 4 Discussion

### 4.1 Comparison with existing estimates

Direct comparisons among existing PFAS emission studies are inherently challenging because available estimates differ in study period, spatial coverage, source categories, activity data, and estimation methodology. Nevertheless, comparisons with previous inventories provide useful context for evaluating the magnitude and temporal trajectory of the present estimates. Xie et al. [21] estimated annual PFOS-equivalent emissions from major industrial sources in China at approximately 70 t yr^−1^ for 2010. Liu et al. [23] reported PFOS and PFOA emissions of 70 and 40 t yr^−1^, respectively, for central and eastern China, with nearly 90% attributed to industrial sources. In comparison, our national estimates for 2016 were 49 t yr^−1^ (40–55 t yr^−1^) for PFOS and 75 t yr^−1^ (62–90 t yr^−1^) for PFOA (Fig. 5b). These differences likely reflect temporal changes in production and use, geographic coverage, sectoral inclusion, and estimation methods.

**Fig. 5:**
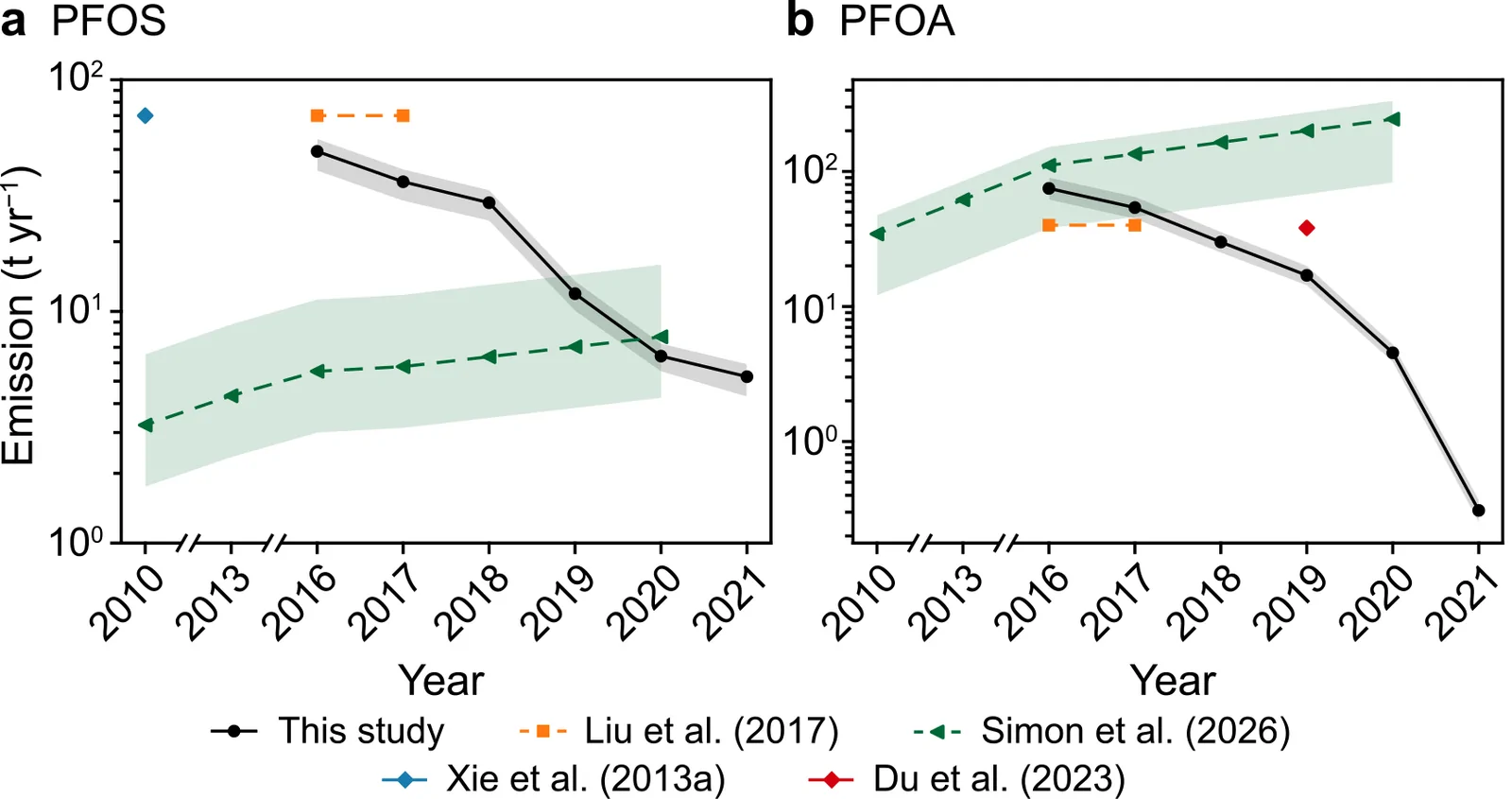
**Comparison of national PFOS and PFOA emission estimates from this study and previously published inventories**. Annual national emission estimates for PFOS (**a**) and PFOA (**b**) from this study and previous inventories. Estimates from Xie et al. [21], Liu et al. [23], Du et al. [29], and Simon et al. [25] are shown for comparison. Shaded bands indicate reported uncertainty ranges where available. PFOS, perfluorooctane sulfonate; PFOA, perfluorooctanoic acid.

Medium-specific PFOA emission estimates differ substantially among inventories (Fig. 5a). Du et al. [29] estimated atmospheric PFOA emissions at approximately 38 t yr^−1^ in 2019, whereas Simon et al. [25] reported a substantially higher estimate of 120 t yr^−1^ for China and continued increases in PFOS and PFOA emissions through 2020. Simon et al. [25] developed a global gridded inventory of PFOS and PFOA emissions from 1950 to 2020, focusing on releases from airport firefighting and product use or disposal for PFOS and from airport firefighting, fluoropolymer production, and product use or disposal for PFOA. Its global scope limited the incorporation of detailed China-specific information, including sector-level production changes, facility closures, technological transitions, and the timing of national restrictions. By contrast, the present inventory incorporates China-specific source activity data and captures the marked reductions estimated for major PFOS- and PFOA-related pathways.

The activity data underlying the present inventory were also compared with independent production statistics. The national estimates of PFOS-related production, including its key precursor PFOSF, compiled in this study showed strong temporal agreement with data reported by Zhang et al. [39] and in the *Annual Environmental Statistics* released by the Ministry of Ecology and Environment of China in 2020 and 2021 [40], [41] (Pearson’s *r* = 0.987, *p* < 0.001; *R*^*2*^ = 0.973; Supplementary Fig. S7 and Table S4).

### 4.2 Consistency with environmental monitoring records

Because PFOS and PFOA are persistent organic pollutants, annual emissions and observed environmental concentrations must be compared cautiously. Emission inventories and environmental monitoring data represent different stages of the source-to-environment continuum. Emission inventories quantify annual releases from identified sources, whereas measured environmental concentrations reflect the cumulative effects of current and historical emissions, multimedia transport and partitioning, transformation, and long-term accumulation. Consequently, annual emission estimates cannot be directly validated through one-to-one comparisons with concentrations measured in soil, water, sediment, or biota. Moreover, temporal lags and spatial displacement may occur between emission sources and observed contamination hotspots [42], [43].

At the temporal scale, the downward trends estimated in this inventory are directionally consistent with several long-term monitoring records from China. PFOS concentrations in agricultural soils in eastern China declined between 2011 and 2021 following regulatory controls and industrial phase-outs [26]. Surface-water monitoring in the Xiaoqing River Basin also showed an overall decrease in total PFAS concentrations, particularly PFOS and PFOA, between 2013 and 2023 [44]. The timing and magnitude of these changes differed among environmental media. These records therefore support only the general direction of the estimated decline and do not provide direct quantitative validation.

At the spatial scale, the broad emission pattern is consistent with published monitoring evidence (Supplementary Fig. S8). The inventory identifies elevated emissions in industrialized eastern and southern China, including eastern coastal zones, the Pearl River Delta, and several northern and central industrial regions. A nationwide survey of 124 background surface-soil samples collected in 2021 similarly reported higher PFAS concentrations in eastern, southern, and coastal China [45]. Regional studies provide further support for specific areas. Measurements of seawater, suspended particulate matter, and sediment in the Pearl River Estuary demonstrated widespread PFAS contamination, with PFOA dominating the seawater phase and industrial activities in the Pearl River Delta identified as important sources [46]. A nationwide synthesis of 41,492 records for 180 legacy and emerging PFAS across eight major river basins also identified comparatively elevated aquatic contamination in the Yellow River and Liaohe River regions [8]. These observations are broadly consistent with the southern, northern, and eastern patterns identified in the inventory. Local differences are expected because monitoring results depend on sampling location, spatial coverage, sampling period, and environmental medium, whereas gridded emissions are partly allocated using facility locations and source-specific spatial proxies. The comparison therefore indicates regional consistency rather than grid-level agreement.

Taken together, available monitoring records are directionally consistent with the estimated national decline and the broad concentration of emissions in industrialized eastern and southern China; however, these comparisons remain qualitative. More rigorous quantitative validation would require high-resolution multimedia fugacity models to simulate environmental concentrations under consistent spatial and temporal conditions.

### 4.3 High-emission zones and spatial patterns of potential exposure

The population-weighted emission index (Supplementary Fig. S9) shows patterns of population–emission overlap that differ from those in the unweighted emission maps. The index multiplies grid-cell emissions by each cell’s share of the national population and provides a screening measure of spatial overlap; it does not quantify exposure or health risk. Both PFOS and PFOA indices exhibited a pronounced east–west contrast broadly aligned with the Heihe–Tengchong Line. The comparatively low population-weighted PFOS values in Jiangsu partly reflect the lower AFFF allocation derived from the fire-incident proxy. YRD and PRD emerged as priority areas where residual emission sources coincided with dense populations. These regions warrant consideration in future concentration modeling, environmental monitoring, and exposure assessment.

As sulfluramid- and AFFF-related emissions declined, the remaining inventory became increasingly dominated by localized industrial sources. Prioritize environmental monitoring and exposure assessment for residual PFOS sources associated with metal plating and firefighting activities, along with PFOA hotspots near fluoropolymer facilities. Toxicological and epidemiological evidence provides a health-related rationale for such analyses [47], [48], [49], [50], [51]; however, the present inventory does not quantify human exposure or health risk.

### 4.4 Strengths, limitations, and future directions

The estimated emission declines coincided with several sector-specific measures implemented during 2016–2021, including the PFOS priority-control listing and phase-out project [20], the 2019 restrictions on PFOS-related substances [18], the 2020 prohibition of sulfluramid use [32], and the PFOA priority-control listing [19] (Supplementary Table S3). This temporal and sectoral correspondence is consistent with a potential influence of source-directed regulation and industrial transition. However, the observational inventory lacks a counterfactual attribution framework and cannot isolate the effects or relative contributions of individual policies, technological changes, market substitution, or changes in industrial activity.

The observed temporal reduction reflects a structural transformation in the national emission profile of legacy C8 PFAS. The emission source composition shifted from geographically dispersed releases associated with sulfluramid application and widespread industrial use toward a greater contribution from localized sources associated with specific manufacturing facilities and firefighting activities. Although current PFOS and PFOA source inputs have declined, residual emissions are increasingly concentrated in industrialized and densely populated regions. Therefore, prioritize targeted control measures for the remaining metal-plating and firefighting-related sources.

Several limitations of this study warrant acknowledgment and highlight future research directions. First, the temporal scope extends only through 2021 because of data availability. Annual PFOA and PFOS emissions could decline to approximately 0.04 and 1 t yr^−1^, respectively, in the near term. However, actual trajectories may differ depending on changes in industrial activity, chemical substitution, and implementation of the Action Plan for New Pollutants Governance. Second, the inventory is limited to the legacy C8 compounds PFOS and PFOA; therefore, the reported declines should not be interpreted as reductions in total PFAS emissions or environmental pollution. The phase-out of PFOS and PFOA has promoted substitution with short-chain perfluoroalkyl acids (PFAAs), such as perfluorobutane sulfonic acid (PFBS) and perfluorobutanoic acid (PFBA), as well as ether-based alternatives, including F-53B (6:2 chlorinated polyfluorinated ether sulfonate, 6:2 Cl-PFESA) and hexafluoropropylene oxide dimer acid (HFPO-DA), a GenX chemical [38], [52]. Increasing F-53B releases have been documented in China following its expanded use in electroplating [53]. Such substitutions may redistribute PFAS emissions among compounds, sectors, and regions rather than eliminate the overall pollution burden [26], [42]. Replacement PFAS were not included in the present inventory because nationally consistent activity data and process-specific emission factors remain unavailable. Future inventories should expand compound coverage as these data become available. Third, substantial uncertainties remain despite the application of Monte Carlo simulation. Several emission factors were derived from earlier Western technical reports and may not represent contemporary industrial practices in China. Advances in process recovery and pollution control may have reduced emissions at some facilities, but the direction and magnitude of any potential bias cannot be quantified with the available data. Future inventory development should prioritize facility-level mass-balance measurements and China-specific process-level emission factors. Sensitivity analysis (Supplementary Fig. S10) indicates that uncertainty is primarily associated with emission factors and activity data. For PFOS in 2016, activity data for metal plating and AFFFs contributed approximately 39% and 32% of total variance, respectively. For PFOA, the emission factor and activity data for fluoropolymer production accounted for 56% and 37%, respectively. Improving inventory reliability will therefore require better activity data and emission factors, with priorities tailored to individual source sectors.

Emission inventories provide a quantitative foundation for multimedia fate and risk assessment. Future studies should couple this inventory with high-resolution multimedia fate, exposure, and toxicokinetic models. Such integration would enable estimation of environmental concentrations, external and internal exposure, and the potential health benefits of source-control strategies, thereby informing future regulatory priorities.

## 5 Conclusions

This study developed nationally consistent, spatially explicit multimedia emission inventories of PFOS and PFOA in the Chinese mainland for 2016–2021. Total PFOS and PFOA emissions declined by approximately 89% and more than 99%, respectively, while the source profile shifted from broadly distributed sources toward a smaller number of localized industrial and firefighting-related emission hotspots. Water remained the dominant receiving medium for both compounds, although their medium-specific source compositions differed. These changes coincided with regulatory restrictions and technological transitions. Population-weighted screening further identified densely populated areas in eastern and southern regions, particularly the Yangtze River Delta and Pearl River Delta, as priorities for future monitoring and exposure assessment. These inventories provide spatially and temporally resolved source terms for multimedia fate modeling and continued evaluation of source-control strategies.

## CRediT authorship contribution statement

**Shuyu Ouyang:** Writing – original draft, Visualization, Methodology, Formal analysis, Data curation. **Min Wu:** Resources, Methodology, Data curation. **Ke Jiang:** Writing – review & editing, Resources, Methodology, Formal analysis. **Rongcan Chen:** Resources, Methodology, Formal analysis. **Ying Zhu:** Writing – review & editing, Validation, Software, Resources, Methodology. **Huizhong Shen:** Visualization, Resources, Methodology, Formal analysis. **Hefa Cheng:** Visualization, Formal analysis, Data curation. **Bin Wang:** Visualization, Validation, Software, Data curation. **Guofeng Shen:** Writing – review & editing, Visualization, Validation, Supervision, Funding acquisition, Formal analysis, Conceptualization. **Shu Tao:** Resources, Methodology, Data curation.

## Data availability

The activity data, emission factor and parameter datasets, gridding proxy data, gridded PFOS and PFOA emission datasets, and calculation codes that support the findings of this study are available from the corresponding author upon reasonable request.

## Declaration of competing interest

The authors declare that they have no known competing financial interests or personal relationships that could have appeared to influence the work reported in this paper.

## Appendix A Supplementary data

Supporting Information is provided as a separate file. Details of source and process in the inventory framework; emission data, and correlation between emissions and the economic conditions at the provincial and city levels.

## References

[1] Buck R.C., Franklin J., Berger U., Conder J.M., Cousins I.T., de Voogt P., Jensen A.A., Kannan K., Mabury S.A., van Leeuwen S.P.J. (2011). Perfluoroalkyl and polyfluoroalkyl substances in the environment: terminology, classification, and origins. Integrated Environ. Assess. Manag..

[2] Evich M.G., Davis M.J.B., McCord J.P., Acrey B., Awkerman J.A., Knappe D.R.U., Lindstrom A.B., Speth T.F., Tebes-Stevens C., Strynar M.J., Wang Z., Weber E.J., Henderson W.M., Washington J.W. (2022). Per- and polyfluoroalkyl substances in the environment. Science.

[3] Wang Z., DeWitt J.C., Higgins C.P., Cousins I.T. (2017). A never-ending story of Per- and polyfluoroalkyl substances (PFASs)?. Environ. Sci. Technol..

[4] Kim S.-K., Kannan K. (2007). Perfluorinated acids in air, rain, snow, surface runoff, and Lakes: relative importance of pathways to contamination of urban Lakes. Environ. Sci. Technol..

[5] Chen S., Jiao X.-C., Gai N., Li X.-J., Wang X.-C., Lu G.-H., Piao H.-T., Rao Z., Yang Y.-L. (2016). Perfluorinated compounds in soil, surface water, and groundwater from rural areas in eastern China. Environ. Pollut..

[6] Lin H., Taniyasu S., Yamazaki E., Wei S., Wang X., Gai N., Kim J.H., Eun H., Lam P.K.S., Yamashita N. (2020). Per- and polyfluoroalkyl substances in the air particles of Asia: levels, seasonality, and size-dependent distribution. Environ. Sci. Technol..

[7] Wang S., Cai Y., Ma L., Lin X., Li Q., Li Y., Wang X. (2022). Perfluoroalkyl substances in water, sediment, and fish from a subtropical river of China: environmental behaviors and potential risk. Chemosphere.

[8] Li M., Wu K., Zhu J., Pang Z., Wu H., Qiu G., Wei C. (2026). Spatiotemporal distribution, source apportionment, and driving factors of per- and polyfluoroalkyl substances in the river-to-sea systems of China between 2004 and 2024. Water Res..

[9] Sunderland E.M., Hu X.C., Dassuncao C., Tokranov A.K., Wagner C.C., Allen J.G. (2019). A review of the pathways of human exposure to poly- and perfluoroalkyl substances (PFASs) and present understanding of health effects. J. Expo. Sci. Environ. Epidemiol..

[10] Pérez F., Nadal M., Navarro-Ortega A., Fàbrega F., Domingo J.L., Barceló D., Farré M. (2013). Accumulation of perfluoroalkyl substances in human tissues. Environ. Int..

[11] Barry V., Winquist A., Steenland K. (2013). Perfluorooctanoic acid (PFOA) exposures and incident cancers among adults living near a chemical plant. Environ. Health Perspect..

[12] Chen L., Tong C., Huo X., Zhang J., Tian Y. (2021). Prenatal exposure to perfluoroalkyl and polyfluoroalkyl substances and birth outcomes: a longitudinal cohort with repeated measurements. Chemosphere.

[13] Habib Z., Song M., Ikram S., Zahra Z. (2024). Overview of Per- and polyfluoroalkyl substances (PFAS), their applications, sources, and potential impacts on human health. Pollutants.

[14] Gaillard L., Barouki R., Blanc E., Coumoul X., Andréau K. (2025). Per- and polyfluoroalkyl substances as persistent pollutants with metabolic and endocrine-disrupting impacts. Trends Endocrinol. Metabol..

[15] Gui S.-Y., Qiao J.-C., Xu K.-X., Li Z.-L., Chen Y.-N., Wu K.-J., Jiang Z.-X., Hu C.-Y. (2023). Association between per- and polyfluoroalkyl substances exposure and risk of diabetes: a systematic review and meta-analysis. J. Expo. Sci. Environ. Epidemiol..

[16] Secretariat of the Stockholm Convention, All POPs listed in the Stockholm convention, Stockholm convention on persistent organic pollutants, https://www.pops.int/tabid/2509/Default.aspx?utm_source=chatgpt.com (accessed 4 September 2026).

[17] (2019). National Development and Reform Commission of the People’s Republic of China:.

[18] Ministry of Ecology and Environment of the People’s Republic of China (2019). Ministry of Ecology and Environment of the People’s Republic of China:.

[19] (2020). Ministry of Ecology and Environment of the People’s Republic of China:.

[20] World Bank Group (2017).

[21] Xie S., Wang T., Liu S., Jones K.C., Sweetman A.J., Lu Y. (2013). Industrial source identification and emission estimation of perfluorooctane sulfonate in China. Environ. Int..

[22] Li L., Zhai Z., Liu J., Hu J. (2015). Estimating industrial and domestic environmental releases of perfluorooctanoic acid and its salts in China from 2004 to 2012. Chemosphere.

[23] Liu Z., Lu Y., Wang P., Wang T., Liu S., Johnson A.C., Sweetman A.J., Baninla Y. (2017). Pollution pathways and release estimation of perfluorooctane sulfonate (PFOS) and perfluorooctanoic acid (PFOA) in central and eastern China. Sci. Total Environ..

[24] Xie S., Lu Y., Wang T., Liu S., Jones K., Sweetman A. (2013). Estimation of PFOS emission from domestic sources in the eastern coastal region of China. Environ. Int..

[25] Simon P., Ramacher M.O.P., Hagemann S., Matthias V., Joerss H., Bieser J. (2026). POPE: an annual global half degree emission inventory for PFAS 1950–2020. Earth Syst. Sci. Data.

[26] Cheng Y., An Q., Qi H., Li R., Liu W., Gu B., Liu K. (2023). Temporal trends of legacy and emerging PFASs from 2011 to 2021 in agricultural soils of eastern China: impacts of the Stockholm convention. Environ. Sci. Technol..

[27] Ma K., Lu Y., Zhang Y., Zhang Y. (2024). Trend of PFAS concentrations and prediction of potential risks in Taihu Lake of China by AQUATOX. Environ. Res..

[28] Yang C.-P., Wu Q.-E., Jia C.-B., Liu Y., Wang L.-M., Shan B.-B., Liu N., Chen C., Liu M.-T., Huang Y.-B., Sun D.-R. (2025). Per- and polyfluoroalkyl substances (PFAS) in urbanized coastal zones: spatiotemporal distribution, phase partitioning dynamics, and risk assessment in Shenzhen, China. Environ. Int..

[29] Du D., Lu Y., Li Q., Zhou Y., Cao T., Cui H., Han G. (2023). Estimating industrial process emission and assessing carbon dioxide equivalent of perfluorooctanoic acid (PFOA) and its salts in China. Sci. Total Environ..

[30] Xu X. (2017). Resource and Environment Science and Data Center.

[31] (2022). General Office of the State Council of the People’s Republic of China:.

[32] (2019). Ministry of Agriculture and Rural Affairs of the People’s Republic of China:.

[33] (2022). Ministry of Ecology and Environment of the People’s Republic of China:.

[34] Ministry of Ecology and Environment of the People’s Republic of China (2023).

[35] Ministry of Environmental Protection of the People’s Republic of China (2014). Ministry of Environmental Protection of the People’s Republic of China:.

[36] Ministry of Environmental Protection of the People’s Republic of China (2017). Ministry of Environmental Protection of the People’s Republic of China:.

[37] Jiang X., Zhou Z., Qin Z., Ou T., Zhang Q., Zhang H., Wu X., He S., Meng B., Ge Y., Huang J., Zhang Y., Peng Z., Yu G., Deng S. (2024). Occurrence, transport, and full-scale adsorptive removal of PFAS in electroplating parks in China. Environ. Sci. Technol..

[38] Wang P., Fang M., Guan Y. (2013). Research progress on the application of PFOA alternatives in fluoropolymers. Zhejiang Chem. Ind..

[39] Zhang L., Zheng Z., Chen W., Ning Y., Chen Z., Wang J., Du T. (2022). Feasibility analysis and recommendations of disposal technology of PFOS/PFOSF-Containing wastes in China. Res. Environ. Sci..

[40] (2020). Ministry of Ecology and Environment of the People’s Republic of China: Beijing.

[41] (2021). Ministry of Ecology and Environment of the People’s Republic of China: Beijing.

[42] O’Rourke E., Losada S., Barber J.L., Scholey G., Bain I., Pereira M.G., Hailer F., Chadwick E.A. (2024). Persistence of PFOA pollution at a PTFE production site and occurrence of replacement PFASs in English freshwaters revealed by sentinel Species, the eurasian otter (Lutra lutra). Environ. Sci. Technol..

[43] Galloway J.E., Moreno A.V.P., Lindstrom A.B., Strynar M.J., Newton S., May A.A., Weavers L.K. (2020). Evidence of air dispersion: HFPO–DA and PFOA in Ohio and West Virginia surface water and soil near a fluoropolymer production facility. Environ. Sci. Technol..

[44] Liu Y., Lei H., Lu Y., Wang P., Wu T. (2025). Multiple impacts of human activities on environmental fate of per- and polyfluoroalkyl substances (PFAS) in the Xiaoqing River of China. Environ. Pollut..

[45] Wang D., Liu X., Guo Z., Shan W., Yang Z., Chen Y., Ju F., Zhang Y. (2024). Legacy and novel Per- and Polyfluoroalkyl substances in surface soils across China: source tracking and main drivers for the spatial variation. Environ. Sci. Technol..

[46] Tang B., Peng C., Zhou D.-J., Wu K.-Y., Tian F., Chen H.-G., Zhang S.-Y., Li B.-W., Hu G.-C., Ren M.-Z., Distribution J. Zheng (2025). Partitioning behaviors, and source identification of legacy and emerging per- and polyfluorinated alkyl substances in the Pearl River Estuary, South China. Water Res..

[47] He L., Liu C., Zhao H., Yang Z., Zhou E., Chen H., Wei H., Luo B. (2025). Unveiling the hidden threat: Per- and Polyfluoroalkyl Substance Pollution’s distinct traits and crucial impacts on lung function in Chinese Plateau populations. Environ. Health.

[48] Choi J., Kim J.-Y., Lee H.-J. (2022). Human evidence of perfluorooctanoic acid (PFOA) exposure on hepatic disease: a systematic review and meta-analysis. Int. J. Environ. Res. Publ. Health.

[49] Ji C., Pavuk M., Ruiz P. (2025). Global serum Per- and Polyfluoroalkyl Substances exposures and their correlation with lipids: a systematic review and meta-analysis. Environ. Sci. Technol..

[50] Zhang B., Li Q., Wang W., Tian M., Xu D., Xie Y. (2025). PFOS and its substitute OBS cause endothelial dysfunction to promote atherogenesis in ApoE–/– mice. Environ. Health.

[51] Zahm S., Bonde J.P., Chiu W.A., Hoppin J., Kanno J., Abdallah M., Blystone C.R., Calkins M.M., Dong G.-H., Dorman D.C., Fry R., Guo H., Haug L.S., Hofmann J.N., Iwasaki M., Machala M., Mancini F.R., Maria-Engler S.S., Møller P., Ng J.C., Pallardy M., Post G.B., Salihovic S., Schlezinger J., Soshilov A., Steenland K., Steffensen I.-L., Tryndyak V., White A., Woskie S., Fletcher T., Ahmadi A., Ahmadi N., Benbrahim-Tallaa L., Bijoux W., Chittiboyina S., de Conti A., Facchin C., Madia F., Mattock H., Merdas M., Pasqual E., Suonio E., Viegas S., Zupunski L., Wedekind R., Schubauer-Berigan M.K. (2024). Carcinogenicity of perfluorooctanoic acid and perfluorooctanesulfonic acid. Lancet Oncol..

[52] Blum A., Balan S.A., Scheringer M., Trier X., Goldenman G., Cousins I.T., Diamond M., Fletcher T., Higgins C., Lindeman A.E., Peaslee G., de Voogt P., Wang Z., Weber R. (2015). The Madrid statement on Poly- and Perfluoroalkyl substances (PFASs). Environ. Health Perspect..

[53] Ti B., Li L., Liu J., Chen C. (2018). Global distribution potential and regional environmental risk of F-53B. Sci. Total Environ..

