# Supplementary material for: Spatiotemporal inventory of PFOS and PFOA in China’s air, water and soil (2016–2021): Sharp declines yet persistent hotspots
Source: Environ Sci Ecotechnol. 2026 Oct 29;34:100785. doi: 10.1016/j.ese.2026.100785 (PMC13755980; doi:10.1016/j.ese.2026.100785)
Supplement: Supplementary file 1 [file mmc1.docx]

**Supplementary Information**

**Spatiotemporal inventory of PFOS and PFOA in China's air, water and soil (2016–2021): Sharp declines yet persistent hotspots**

Shuyu Ou’yang^a^, Min Wu^a, ‡^, Ke Jiang^a^, Rongcan Chen^a^, Ying Zhu^b,c^, Huizhong Shen^d,e^, Hefa Cheng^a^, Bin Wang^a,f^, Guofeng Shen^a,g,h *^, Shu Tao^a,d^

a. College of Urban and Environmental Sciences, Peking University, Beijing 100871, China

b. State Environmental Protection Key Laboratory of Environmental Health Impact Assessment of Emerging Contaminants, Shanghai Jiao Tong University, Shanghai 200240, China

c. School of Environmental Science and Engineering, Shanghai Jiao Tong University, Shanghai 200240, China

d. School of Environmental Science and Engineering, Southern University of Science and Technology, Shenzhen 518055, China

e. Guangdong Provincial Observation and Research Station for Coastal Atmosphere and Climate of the Greater Bay Area, Shenzhen 518055, China

f. Institute of Reproductive and Child Health, Peking University, Beijing 100191, China

g. Institute of Carbon Neutrality, Peking University, Beijing 100871, China

h. Center for Environment and Health, Peking University, Beijing 100871, China

‡ Present address: Department of Civil and Environmental Engineering, The Hong Kong Polytechnic University, Hong Kong, China

* Corresponding author: College of Urban and Environmental Sciences, Peking University, Email: gfshen12@pku.edu.cn; Tel: 86-10-62753293

**There are 1 text, 4 tables and 8 figures in this appendix**

**Table of Contents**

[Text S1. Emission Inventory: Detailed Methods and Data Sources	1](#_Toc26664)

[S1.1 PFOS Emission Sources	1](#_Toc9787)

[S1.1.1 PFOS Manufacturing	1](#_Toc31198)

[S1.1.2 Metal Electroplating (Chrome Mist Suppressant)	1](#_Toc6415)

[S1.1.3 Aqueous Film-Forming Foams (AFFFs) — Production	2](#_Toc25121)

[S1.1.4 Aqueous Film-Forming Foams (AFFFs) — Use	2](#_Toc29735)

[S1.1.5 Semiconductor Manufacturing (Photoresist)	3](#_Toc4816)

[S1.1.6 Sulfluramid Production	4](#_Toc9046)

[S1.1.7 Sulfluramid Use	4](#_Toc11317)

[S1.2 PFOA Emission Sources	5](#_Toc8818)

[S1.2.1 PFOA Manufacturing	5](#_Toc3232)

[S1.2.2 AFFF Containing PFOA — Production and Use	5](#_Toc10519)

[S1.2.3 Sulfluramid Use (PFOA Component)	5](#_Toc15773)

[S1.2.4 Fluoropolymer (FP) Manufacturing	6](#_Toc4475)

[Table S1. Emission factors (EFs) for PFOS and PFOA from various source categories.	7](#_Toc13308)

[Table S2. Temporal Changes in the Relative Variability Index (RVI) of grid-level PFOS and PFOA emissions.	9](#_Toc15068)

[Table S3. Key PFOS- and PFOA-related regulatory and policy milestones relevant to the 2016–2021 inventory and the corresponding emission trends observed in this study.	10](#_Toc16650)

[Table S4. Quantitative comparison of national PFOS/PFOSF production estimates from this study and the independent dataset during the overlapping period of 2016–2021.	12](#_Toc27718)

[Fig. S1. Distribution of reported fire incidents across AFFF-relevant application scenarios in China in 2021.	13](#_Toc18094)

[Fig. S2. Spatial distributions and statistical characteristics of annual PFOA and PFOS emission densities in the Chinese mainland in 2016 and 2021. 	15](#_Toc8914)

[Fig. S3. Spatial Distributions of Absolute and Relative Emission Reductions	16](#_Toc3395)

[Fig. S4. Temporal Trends of Medium-Specific Emissions	17](#_Toc20103)

[Fig. S5. Spatial Distributions of Medium-Specific Emissions	18](#_Toc20837)

[Fig. S6. Exploratory bivariate relationships between city-level PFOS and PFOA emissions and total, industrial, and agricultural GDP.	19](#_Toc23421)

[Fig. S7. Comparison of Estimated PFOS Production with Independent Statistics	21](#_Toc4367)

[Fig. S8. Comparison of the spatial distribution of PFOS and PFOA emissions estimated in this study with previously reported environmental monitoring results in China.	22](#_Toc4348)

[Fig. S9. Spatial distributions of population-weighted emission index for PFOA (a,c) and PFOS (b,d) in 2016 (a,b) and 2021 (c,d).	23](#_Toc6840)

[Fig. S10. Sensitivity Analysis	24](#_Toc24234)

[References for Supplementary Information	25](#_Toc13909)

# Text S1. Emission Inventory: Detailed Methods and Data Sources

This section provides detailed descriptions of the activity data (AD), emission factors (EF), and spatial allocation procedures used to construct the high-resolution national emission inventories of PFOS and PFOA for 2016–2021. All emission factors are summarised in Table S1. Emissions were spatially disaggregated to a 10 km × 10 km grid (~0.1° × 0.1°). For Monte Carlo uncertainty analysis, EFs with reported ranges were modelled as Uniform distributions; those without explicit ranges were assigned Lognormal distributions with a conservative coefficient of variation (CV) of 0.1. For multi-media partitioning EFs, a normalisation algorithm was applied to ensure their sum equals unity. Descriptions of regulatory and technological developments in the source-specific sections are provided as contextual information. Unless explicitly represented in the activity data or emission factors, their individual effects were not parameterized separately and should not be interpreted as formal causal attribution.

## S1.1 PFOS Emission Sources

### S1.1.1 PFOS Manufacturing

Electrochemical fluorination (ECF) is the principal industrial route for producing PFOS and its related substances. In this process, organic precursors are dissolved in liquid anhydrous hydrogen fluoride (HF) and subjected to direct-current electrolysis, causing all hydrogen atoms to be replaced by fluorine. The key intermediate perfluorooctane sulfonyl fluoride (PFOSF) is subsequently converted to a range of PFOS products through hydrolysis, amidation, and quaternization reactions. This process is analogous to that formerly employed by the global manufacturer 3M, and comparable emission scenarios are therefore applicable to Chinese production facilities [S1].

Annual PFOS production quantities (Q(production)) were obtained from industry surveys. Atmospheric and aqueous emissions were estimated as:

*Emi(production,air)	= Q(production) × EF(production,air)*	(EqS1)

*Emi(production,water) = Q(production) × EF(production,water)*	(EqS2)

Emission factors (Table S1) are adopted from the 3M voluntary use and exposure information profile [S1]. Grid allocation was based on the geographic coordinates of individual production facilities identified through the industry survey.

### S1.1.2 Metal Electroplating (Chrome Mist Suppressant)

PFOS-based substances have been widely used as mist suppressants in decorative and hard chrome electroplating. In chrome plating baths, PFOS reduces the surface tension of the electrolyte, facilitating the escape of hydrogen and oxygen gas bubbles and forming a dense foam layer on the bath surface, which effectively suppresses chromate aerosol generation and reduces raw material losses without affecting the physical properties or corrosion resistance of the deposited layer [S2]. Although classified as a non-consumable additive, PFOS is continuously removed from the bath via drag-out on workpiece surfaces.

Prior to 26 March 2019, China imposed no specific treatment requirements on PFOS-containing electroplating wastewater, leading to direct environmental discharge. Following the joint announcement by 11 ministries (including the Ministry of Ecology and Environment and the Ministry of Industry and Information Technology) [S3], PFOS use in metal plating was restricted to closed-loop systems in which plating solutions and rinse water are recycled internally with no external discharge. Following the 2019 announcement, PFOS use in hard-metal plating was permitted only in closed-loop systems. However, facility-specific retrofit dates and abatement efficiencies were unavailable. Therefore, no additional plant-level removal factor associated with closed-loop operation was applied in the inventory. The estimated temporal changes primarily reflect the available activity data, while the independent effect of closed-loop retrofits was not quantified.

The primary pathway of PFOS release to water is drag-out of surface liquid on workpieces; a minor fraction is released to air as acid mist from the plating bath [S2]. Provincial PFOS consumption in electroplating (Q(plating)) was apportioned to provinces using electroplated steel output ratios (R(plating)) from China Iron and Steel Industry Yearbooks [4]; grid allocation used enterprise staffing size as a spatial proxy.

*Emi(plating,air)	= Q(plating) × EF(plating,air)	× R(plating)*	(EqS3)

*Emi(plating,water) = Q(plating) × EF(plating,water) × R(plating)*	(EqS4)

### S1.1.3 Aqueous Film-Forming Foams (AFFFs) — Production

Owing to its outstanding thermal stability, chemical stability, and surface-tension-reducing properties, PFOS has been incorporated into aqueous film-forming foams (AFFFs) as a key fluorinated surfactant. AFFFs reduce the surface tension of the firefighting foam, enhancing its flowability and oil-repellency and thereby improving extinguishing efficiency. In China, PFOS-containing AFFFs are not used in ordinary civil or commercial fire suppression; they are deployed primarily in the petrochemical sector, professional fire brigades, and military contexts (e.g., field fuel depots and vessels), where the nature of the fire hazard demands high-performance suppression agents. China ceased PFOS production by the end of 2021.

AFFF production generates alkaline wastewater, acid rinsing water, process effluents, and raw-material losses, with phenolic preservatives, surfactants, and added PFOS/PFOA as the main contaminants. Emissions were estimated from PFOS consumption in AFFF formulation (Q(AF)) using media-specific EFs from Table S1, adopted from the EU Technical Guidance Document (TGD) [S5]. Grid allocation was based on AFFF facility locations identified in the industry survey.

*Emi(AFP,air)	= Q(AF) × EF(AFP,air)*	(EqS5)

*Emi(AFP,water) = Q(AF) × EF(AFP,water)*	(EqS6)

*Emi(AFP,soil) = Q(AF) × EF(AFP,soil)*	(EqS7)

### S1.1.4 Aqueous Film-Forming Foams (AFFFs) — Use

Under typical Chinese firefighting practices, no containment or collection measures are applied to spent foam; all PFOS in deployed AFFFs is therefore assumed to be released directly to the environment, with 50% partitioned to water and 50% to soil [S6]. Annual AFFF consumption was estimated at 24.6% of total AFFFs production (utilisation factor F(U)) based on a literature survey of Chinese firefighting practice. Provincial-level emissions were apportioned by each province's share of national annual fire incidents (R(fire)) from China Statistical Yearbooks [S7-S12].

Province-level AFFF-use emissions to water and soil were calculated as:

*Emi(AFU,water) = Q(AF) × F(U) × EF(AFU,water) × R(fire)*	(EqS8)

*Emi(AFU,soil) = Q(AF) × F(U) × EF(AFU,soil) × R(fire)*	(EqS9)

where *Q(AF)* is the national AFFF production, and *R(fire)* is the share of national fire incidents occurring in province p.

AFFF is primarily used to suppress high-hazard Class B fires involving flammable liquids. Documented application settings include petroleum refineries, chemical plants, bulk fuel-storage facilities, gasoline filling stations, airports, maritime operations, military installations, and specialized firefighting services [S40]. Fire-by-place statistics from the *China Fire and Rescue Yearbook* [S41-S44] showed that petrochemical production units, tank farms, and gasoline filling stations accounted for 96.2% of the selected AFFFs-relevant fire incidents **(Fig. S1)**. Therefore, province-level AFFFs-use emissions were spatially allocated to petrochemical and chemical facility points and gasoline filling-station points according to their normalized fire-incident proportions [S45]. In the absence of nationally consistent information on facility-level AFFF stocks, actual AFFF use, fuel-storage capacity, or fire-protection-system scale, emissions were distributed equally among facilities within each category and province. If no petrochemical or chemical facility point was identified in a province, the corresponding allocation share was reassigned to gasoline filling stations within the same province to preserve the provincial emission total. The fire-incident proportions were used only as spatial-allocation proxies and do not imply that AFFF was deployed in every reported incident.

### S1.1.5 Semiconductor Manufacturing (Photoresist)

In semiconductor fabrication, photoresist is applied to a substrate and selectively exposed through a photomask; exposed or unexposed regions are dissolved during development to transfer circuit patterns to the substrate via etching. PFOS-based substances serve as surfactants to promote uniform photoresist coating, and as photoacid generators (PAGs) and anti-reflective coating (ARC) components in high-end photoresist formulations [S2]. PFOS use is concentrated in integrated circuit (IC) manufacturing rather than discrete device fabrication. In China, the domestic market is dominated by low-end printed-circuit-board (PCB) photoresist; high-end semiconductor photoresist accounts for only approximately 1.6% of the market [S2]. Accordingly, only IC manufacturing is considered as a PFOS source in this inventory [S14].

Based on a mass-balance assessment by the European Semiconductor Industry Association (ESIA) and SEMI, approximately 53.3% of PFOS consumed in semiconductor manufacturing is released to wastewater [S6]. Provincial PFOS consumption in semiconductor use (Q(semicon)) was allocated to provinces using each province's share of national IC output (R(semicon)) from China Statistical Yearbooks [S15-S20]; grid allocation used enterprise size and geographic coordinates of IC manufacturers.

*Emi(semicon,water) = Q(semicon) × EF(semicon,water) × R(semicon)*	(EqS10)

### S1.1.6 Sulfluramid Production

Sulfluramid (N-ethyl perfluorooctane sulfonamide, Et-FOSA) is the sole PFOS-related pesticide registered in China. As an insect energy-metabolism inhibitor, it is formulated primarily as a bait for the control of red imported fire ants (RIFA), cockroaches, termites, and other ant species [S21]. Sulfluramid has held a significant market share in Chinese RIFA prevention and control. In accordance with amendments to the Stockholm Convention on Persistent Organic Pollutants (POPs), the Ministry of Agriculture and Rural Affairs of China (Announcement No. 148) [S22] cancelled pesticide registrations and production licences for all sulfluramid-containing products from 26 March 2019, with use prohibited from 1 January 2020.

Sulfluramid technical material is synthesised from PFOSF, ethylamine, hydrochloric acid, and related solvents. PFOS-equivalent emissions from the production process were calculated using PFOSF consumption (Q(sul,POSF)) and media-specific EFs from Table S1, adopted from the European Chemicals Bureau (ECB/IHCP) Technical Guidance Document [S5]. Emissions were allocated to the geographic coordinates of production facilities identified in the industry survey.

*Emi(sul_prod,air)	= Q(sul,POSF) × EF(sul_prod,air)*	(EqS11)

*Emi(sul_prod,water) = Q(sul,POSF) × EF(sul_prod,water)*	(EqS12)

*Emi(sul_prod,soil) = Q(sul,POSF) × EF(sul_prod,soil)*	(EqS13)

### S1.1.7 Sulfluramid Use

The ultimate metabolite of sulfluramid in the environment is PFOS, posing significant risks to ecosystem health and human health [S23, S24]. Commercial sulfluramid purity (C(sul), Et-FOSA content) and the Et-FOSA to PFOS transformation factor (F(sul)) are listed in Table S1. Given it s relatively low price and hazardous classification, sulfluramid was assumed to be sold and applied in the year of production. Upon application and disposal, 90% of emissions enter soil and 10% are released to air [S21], reflecting the predominant use of bait formulations in terrestrial habitats.

Provincial-level emissions were apportioned using each province's share of national pesticide consumption (R(pesticide)) from China Rural Statistical Yearbooks [25-30]. Grid allocation within each province used farmland and woodland raster data (1 km resolution, resampled to 10 km) from the *China Land Use Remote Sensing Monitoring Dataset (CNLUCC)* [S31].

*Emi(sul_use,air) = Q(sul) × C(sul) × F(sul) × EF(sul_use,air) × R(pesticide)*	(EqS14)

*Emi(sul_use,soil) = Q(sul) × C(sul) × F(sul) × EF(sul_use,soil) × R(pesticide)*	(EqS15)

## S1.2 PFOA Emission Sources

### S1.2.1 PFOA Manufacturing

PFOA and its related substances are produced industrially by two main routes: ECF and fluoroalkene telomerization. In China, ECF is the dominant manufacturing process, yielding PFOA products with a purity of approximately 95% and an isomeric profile analogous to those formerly produced by 3M [S32, S33]. During synthesis and manufacturing, PFOA is released primarily as tail gas, process wastewater, and solid waste [S1]. Annual production quantities (Q(production)) were obtained from industry surveys; EFs for air, water, and soil are listed in Table S1 [S1]. Grid allocation was based on facility locations from the industry survey.

*Emi(production,air)	= Q(production) × EF(production,air)*	(EqS16)

*Emi(production,water) = Q(production) × EF(production,water)*	(EqS17)

*Emi(production,soil) = Q(production) × EF(production,soil)*	(EqS18)

### S1.2.2 AFFF Containing PFOA — Production and Use

PFOA, like PFOS, has been used as a fluorinated surfactant in AFFFs because of its ability to form an aqueous film that suppresses oil-fire vapours through a dual foam-and-film extinguishing mechanism. PFOA-based AFFFs share identical production processes and use conditions with PFOS-based AFFFs described in Sections S1.1.3–S1.1.4. Emissions were estimated following Equations S5–S9 using PFOA-specific activity data (Q(AF)) and EFs from Table S1. Provincial allocation for use-phase emissions was proportional to fire incident frequency (R(fire)), and corresponding facility point datasets were used for spatial allocation [S41-S44].

### S1.2.3 Sulfluramid Use (PFOA Component)

Sulfluramid degrades in the environment via photolysis, oxidation, and biotransformation to yield both PFOS and PFOA as terminal metabolites; PFOA impurities present in the commercial product also enter environmental media upon application [S24]. Two PFOA source terms are therefore considered: (i) PFOA impurity content in the sulfluramid formulation (R(content)); and (ii) transformation of PFOS-precursor material to PFOA at a mean conversion rate R(trans). Both parameters are listed in Table S1. The production stage of sulfluramid does not generate PFOA emissions; only use-phase releases are calculated.

Spatial allocation followed the procedure described in Section S1.1.7, using paddy field and dryland raster data from CNLUCC [31].

*Emi(sul_use,air) = Q(sul)×C(sul)×F(sul)×EF(sul_use,air) ×(R(content)+R(trans))×R(pesticide)*	(EqS23)

*Emi(sul_use,soil) = Q(sul)×C(sul)×F(sul)×EF(sul_use,soil)×(R(content)+R(trans))×R(pesticide)*	(EqS24)

### S1.2.4 Fluoropolymer (FP) Manufacturing

Fluoropolymers such as polytetrafluoroethylene (PTFE), polyvinylidene fluoride (PVDF), and fluorinated ethylene propylene (FEP) exhibit exceptional chemical stability, weathering resistance, corrosion resistance, and antioxidant properties, and are widely used in coatings, industrial components, water treatment systems, electronics, and power generation equipment. PFOA is employed as an emulsifier (processing aid) in the suspension and emulsion polymerisation of backbone-fluorinated polymers and fluoroelastomers, promoting uniform dispersion of the fluoropolymer particles in the reaction medium [S34]. During manufacturing, gaseous PFOA is released via heating and drying operations, while liquid- and solid-phase PFOA enters the environment through dewatering effluents and solid waste [S35].

Following the U.S. Environmental Protection Agency's call for voluntary PFOA phase-out, major international fluorochemical manufacturers (including 3M and DuPont) achieved a 95% reduction from 2000 levels by 2010 and targeted full elimination by 2015. Chinese manufacturers began adopting alternative emulsifiers (e.g., perfluoropolyether-based surfactants) and improving PFOA recovery and recycling at a later stage. Activity data (Q(FP)) and EFs (Table S1) were adopted from Wang et al. (2014) [S36]. Plant- and year-specific information on PFOA recovery efficiency, alternative-emulsifier adoption, and waste-management performance was unavailable. These technological changes are therefore discussed as industrial context and were not parameterized separately, except insofar as they were reflected in the available activity data.

Grid allocation was based on FP facility locations identified in the industry survey.

*Emi(FP,air)	= Q(FP) × EF(FP,air)*	(EqS25)

*Emi(FP,water) = Q(FP) × EF(FP,water)*	(EqS26)

*Emi(FP,soil) = Q(FP) × EF(FP,soil)*	(EqS27)

# Table S1. Emission factors (EFs) for PFOS and PFOA from various source categories.

| **Compound** | **Stage** | **Source Category** | **Reported form** | **EFair** | **EFwater** | **EFsoil** | **Reference** |
| --- | --- | --- | --- | --- | --- | --- | --- |
| **PFOS** | Production | PFOS manufacture | media-specific EF | 0.0045–0.0060 | 0.0023–0.0151 | — | Brooke et al., 2004 [S6] |
|  |  | AFFFs manufacture | media-specific EF | 0.001 | 0.02 | 0.0001 | EC, 2003 [S5] |
|  |  | Sulfluramid manufacture | media-specific EF | 0.0025 | 0.002 | 0.0001 | EC, 2003 [S5] |
|  | Use | Chrome plating | partitioning fraction | 0.002 | 0.998 | — | Xie et al., 2013 [S2] |
|  |  | Firefighting AFFFs use | partitioning fraction | — | 0.5 | 0.5 | Brooke et al., 2004 [S6] |
|  |  | Sulfluramid use | partitioning fraction | 0.1 | — | 0.9 | Wang, 2017 [S21] |
|  |  | Semiconductor photoresist | media-specific EF | — | 0.533 | — | Brooke et al., 2004 [S6] |
| **PFOA** | Production | PFOA manufacture | media-specific EF | 0.002 | 0.041 | 0.00045 | 3M Company, 2003 [S1] |
|  |  | AFFFs manufacture | media-specific EF | 0.001 | 0.02 | — | EC,2003 [S5] |
|  |  | Fluoropolymer processing | media-specific EF | 0.08–0.12 | 0.28–0.42 | 0.04–0.06 | Wang, 2014 [S36] |
|  | Use | Firefighting AFFFs use | partitioning fraction | — | 0.5 | 0.5 | Brooke et al., 2004 [S6] |
|  |  | Sulfluramid use | partitioning fraction | 0.1 | — | 0.9 | Wang, 2017 [S21] |

| **Compound** | **Stage** | **Source Category** | **Reported Form** | ***C(sul)*** | ***R(content)*** | ***R(trans)*** | **Reference** |
| --- | --- | --- | --- | --- | --- | --- | --- |
| **PFOS** | Use | Sulfluramid use | — | 97% (95–99%) | 0 | 95% |  |
| **PFOA** | Use | Sulfluramid use | — | 97% (95–99%) | 556 ppm (200–1600 ppm) | 0.159 (0.05–0.268) | Wang, 2014 [S36] |

Note: "—" indicates no significant emission to the medium. EF values may be dimensionless partitioning fractions or mass-based factors depending on the original source. Although some EFs originate from early technical reports, no comprehensive contemporary China-specific alternatives were identified; these values were therefore retained as baseline estimates, with their reported or assumed uncertainty ranges propagated in the Monte Carlo analysis. For Monte Carlo uncertainty analysis, EFs with reported ranges were modelled as Uniform distributions; those without explicit ranges were assigned Lognormal distributions (CV = 0.1). A normalisation algorithm was applied to multi-media partitioning EFs to ensure their sum equals unity.

# Table S2. Temporal Changes in the Relative Variability Index (RVI) of grid-level PFOS and PFOA emissions.

The Relative Variability Index (RVI) quantifies the degree of spatial heterogeneity in emission distributions. RVI values ranged from 2.05 to 6.36, remaining high in 2016–2019 and declining in 2020 before a partial rebound in 2021., without converging toward unity, indicating persistent spatial heterogeneity.

| **substance** | **year** | **IQR** | **Median** | **RVI** |
| --- | --- | --- | --- | --- |
| **PFOA** | **2016** | 255 | 41.4 | 6.15 |
|  | **2017** | 252 | 45.4 | 5.55 |
|  | **2018** | 218 | 36.8 | 5.92 |
|  | **2019** | 87 | 14.4 | 6.00 |
|  | **2020** | 108 | 52.5 | 2.05 |
|  | **2021** | 62 | 21.1 | 2.92 |
| **PFOS** | **2016** | 1475 | 231.8 | 6.36 |
|  | **2017** | 1460 | 255.6 | 5.71 |
|  | **2018** | 1277 | 206.4 | 6.19 |
|  | **2019** | 485 | 80.8 | 6.00 |
|  | **2020** | 882 | 398.8 | 2.21 |
|  | **2021** | 898 | 331.7 | 2.71 |

# Table S3. Key PFOS- and PFOA-related regulatory and policy milestones relevant to the 2016–2021 inventory and the corresponding emission trends observed in this study.

Broader national policies concerning persistent organic pollutants and hazardous chemicals provided the general governance context but were not included because they did not specify PFOS/PFOA-related sectors or compound-specific measures.

| **Year** | **Policy or action** | **Regulated PFAS** | **Targeted sectors/pathways and principal measures** | **Trends in this study** |
| --- | --- | --- | --- | --- |
| 2009; effective in China in 2014 | Stockholm Convention Annex B amendment and China’s implementation announcement [S46, S47] | PFOS, its salts, and PFOSF | Prohibited production, distribution, use, import, and export except for specified exemptions and acceptable purposes; required substitution and risk control for remaining uses. | / |
| 2017; implementation continued through 2023 | List of Priority Controlled Chemicals (First Batch) and China PFOS Priority Sectors Reduction and Phase-Out Project [S48–S50] | PFOS, its salts, and PFOSF | Targeted PFOS production, electroplating, firefighting foam, and pest control through production phase-out, cleaner production, substitution, supply tracking, technical assistance, and demonstration projects. | PFOS decreased |
| 2019 | Announcement on Prohibiting the Production, Distribution, Use, Import, and Export of Lindane and Other Persistent Organic Pollutants[S3] | PFOS, its salts, and PFOSF | From 26 March 2019, prohibited production, distribution, use, import, and export outside listed acceptable purposes, including closed-loop hard-metal plating and firefighting foam. | PFOS production decreased to zero; only metal plating and AFFFs-related emission remained |
| 2019; effective in 2020 | Guidance Catalogue for Industrial Structure Adjustment (2019 Edition)[S51] | PFOS/PFOSF and PFOA | Restricted new PFOS/PFOA production capacity, classified non-acceptable PFOS products as obsolete, and encouraged alternatives and substitution technologies. | PFOS decreased |
| 2019–2020 | Ministry of Agriculture and Rural Affairs Announcement No. 148 [S22] | Sulfluramid, a PFOS precursor | Stopped new and renewed registrations, revoked registrations and production licences from 26 March 2019, and prohibited use from 1 January 2020. | Sulfluramid-related emissions declined sharply after 2019 and were set to zero in the inventory from 2020 |
| 2019; 2022 | Stockholm Convention Annex A listings [S46] | PFOA, its salts and related compounds; PFHxS, its salts and related compounds | Established international elimination obligations: PFOA with specific exemptions and PFHxS without exemptions; corresponding domestic controls were introduced in the 2023 list. | PFOA decreased |
| 2020 | List of Priority Controlled Chemicals (Second Batch) [S52] | PFOA, its salts, and PFOA-related compounds | Prioritized cross-sector environmental-risk management through restriction, cleaner-production auditing, information disclosure, substitution, and emission-control instruments. | PFOA decreased sharply |
| 2022 | Action Plan for New Pollutants Control[S53] | Priority new pollutants, including convention-listed PFAS | Established a lifecycle framework covering information surveys, monitoring, risk assessment, priority listing, source restrictions, substitution, emission and waste control, and enforcement. | / |
| 2023 | List of New Pollutants for Priority Control (2023 Version) [S54] | PFOS class, PFOA class, and PFHxS class | Banned PFOS production and ended its firefighting-foam exemption in 2023; restricted PFOA to specified uses; prohibited PFHxS without exemptions; also required trade controls, cleaner-production audits, and waste and soil-risk management. | / |

Note: The emission trends reported in this table are direct outputs of the inventory, whereas the links to regulatory and industrial developments indicate temporal and sectoral correspondence only. No counterfactual or formal causal-attribution analysis was conducted. The 2021 draft document is included as a policy-formulation signal, and the 2022–2023 instruments are included solely as post-study governance context.

# Table S4. Quantitative comparison of national PFOS/PFOSF production estimates from this study and the independent dataset during the overlapping period of 2016–2021.

| **Year** | **Independent dataset [S37- S39]**  **(t/a)** | **This study (t/a)** | **Absolute difference (t/a)** | **Relative difference (%)** |
| --- | --- | --- | --- | --- |
| **2016** | 111.72 | 123.86 | 12.14 | 10.87 |
| **2017** | 90.38 | 101.45 | 11.07 | 12.25 |
| **2018** | 69.04 | 93.19 | 24.15 | 34.98 |
| **2019** | 40.17 | 40.61 | 0.44 | 1.10 |
| **2020** | 22.00 | 25.81 | 3.81 | 17.32 |
| **2021** | 12.00 | 20.09 | 8.09 | 67.42 |

# Fig. S1. Distribution of reported fire incidents across AFFF-relevant application scenarios in China in 2021.

Among the 208 selected incidents, petrochemical production units and tank farms accounted for 153 incidents (73.6%), gasoline filling stations for 47 (22.6%), vessels for 5 (2.4%), and aircraft-related incidents for 3 (1.4%). These proportions were used as relative weights for spatially allocating AFFF-use emissions and do not indicate that AFFF was necessarily deployed in every incident. [S41-S44]

# Fig. S2. Spatial distributions and statistical characteristics of annual PFOA and PFOS emission densities in the Chinese mainland in 2016 and 2021.

Panels (a, b) show PFOA, and panels (c, d) show PFOS. Maps use logarithmic color scales, and red rectangles indicate representative high-emission regions. Histograms show the percentage distribution of non-zero-emission grid cells across emission-density intervals. Boxplots show the minimum, quartiles, median, and maximum of all non-zero grid-cell values.

# Fig. S3. Spatial Distributions of Absolute and Relative Emission Reductions

Spatial distributions of both absolute emission reductions and relative reduction ratios for PFOS and PFOA across China during 2016–2021. (a)maps of absolute emission reduction for PFOS and PFOA in 2017 and 2021; (b)maps of relative emission reduction for PFOS and PFOA in 2017 and 2021

(a)

(b)

# Fig. S4. Temporal Trends of Medium-Specific Emissions

Stacked area charts showing the temporal evolution (2016–2021) of PFOS and PFOA emissions disaggregated by environmental medium (water, air, and soil), illustrating the relative contributions of each medium and their changes over time.

# Fig. S5. Spatial Distributions of Medium-Specific Emissions

Gridded spatial distributions of media-specific PFOS and PFOA areal emission intensities in China (2016) with corresponding histograms. (a) PFOS emissions to air. (b) PFOS emissions to water. (c) PFOS emissions to soil. (d) PFOA emissions to air. (e) PFOA emissions to water. (f) PFOA emissions to soil. Notes: Units: mg km-2 yr-1. Grid resolution: 0.1° × 0.1°. Zero-emission grids are masked.

# Fig. S6. Exploratory bivariate relationships between city-level PFOS and PFOA emissions and total, industrial, and agricultural GDP.

Scatter plots showing positive correlations between city-level PFOS and PFOA emission totals and economic activity indicators at the prefecture level. Points represent individual cities; solid lines indicate the regression fit. Pearson correlation coefficients are shown to describe the direction and magnitude of statistical co-variation. These relationships are descriptive and do not represent causal effects or adjusted associations.

|  |  |
| --- | --- |
|  |  |
|  |  |
|  |  |

# Fig. S7. Comparison of Estimated PFOS Production with Independent Statistics

Comparison of national PFOS production totals compiled in this study with independently reported statistics from Zhang (2022) [S37] and the Annual Environmental Statistics of the Ministry of Ecology and Environment of China (2020, 2021) [S38, S39]. Exact numbers in this figure are listed in Table S3.

# Fig. S8. Comparison of the spatial distribution of PFOS and PFOA emissions estimated in this study with previously reported environmental monitoring results in China.

Panels (a) and (b) show nationwide monitoring-based spatial patterns and the major hotspot regions identified at the national scale.[S55,S56] Panel (c) presents a localized but noteworthy hotspot characterized by particularly high measured PFOS and PFOA concentrations. [S57] The original figures were reproduced or adapted from S55-S57.

# Fig. S9. Spatial distributions of population-weighted emission index for PFOA (a,c) and PFOS (b,d) in 2016 (a,b) and 2021 (c,d).

The index equals the annual emission in each grid cell multiplied by the cell’s share of the national population. Values are displayed on logarithmic color scales.

# Fig. S10. Sensitivity Analysis

# References for Supplementary Information

S1.	3M. *Environmental and Health Assessment of Perfluorooctane Sulfonic Acid and Its Salts*. 3M Company; 2003 (DHHS publication no.: Report Number)| (GPO o. Document Number)|.

S2.	Xie S, Wang T, Liu S *et al.* Industrial source identification and emission estimation of perfluorooctane sulfonate in China. *Environ Int*. 2013; **52**: 1-8. doi: 10.1016/j.envint.2012.11.004

S3.	Ministry of Ecology and Environment of the People’s Republic of China. Announcement on Prohibiting the Production, Distribution, Use, Import, and Export of Lindane and Other Persistent Organic Pollutants [in Chinese]. 2019.

S4.	Editorial Committee of China Steel Industry Yearbook. Chapter 6. *China Steel Industry Yearbook*. Beijing: China Metallurgical Industry Press; 2016.

S5.	European Commission. *Technical guidance document in support of Commission Directive 93/67/EEC on risk assessment for new notified substances, Commission Regulation (EC) No. 1488/94 on risk assessment for existing substances and Directive 98/8/EC of the European Parliament and of the Council concerning the placing of biocidal products on the market*. Brussels, Belgium: Joint Research Centre; European Chemicals Bureau; 2003 (DHHS publication no.: Report Number)| (GPO o. Document Number)|.

S6.	Brooke D, Footitt A, Nwaogu TA. *Environmental Risk Evaluation Report: Perfluorooctane Sulphonate (PFOS)*. Rotherdam: Building Research Establishment Ltd; Risk and Policy Analysts Ltd; 2004 (DHHS publication no.: Report Number)| (GPO o. Document Number).

S7.	National Bureau of Statistics. China Statistical Yearbook. *Chapter 8*. Beijing: China Statistics Press; 2017.

S8.	National Bureau of Statistics. China Statistical Yearbook. *Chapter 8*. Beijing: China Statistics Press; 2018.

S9.	National Bureau of Statistics. China Statistical Yearbook. *Chapter 8*. Beijing: China Statistics Press; 2019.

S10.	National Bureau of Statistics. China Statistical Yearbook. *Chapter 8*. Beijing: China Statistics Press; 2020.

S11.	National Bureau of Statistics. China Statistical Yearbook. *Chapter 8*. Beijing: China Statistics Press; 2021.

S12.	National Bureau of Statistics. China Statistical Yearbook. *Chapter 8*. Beijing: China Statistics Press; 2022.

S13.	Xu X. China Population Spatial Distribution Kilometer Grid Dataset. In: (RESDC) RaESaDC (ed.). Beijing: Resource and Environment Science and Data Center (RESDC); 2017.

S14.	Liu C. Preliminary Assessment of PFOS in China and Environmental Risk Assessment of PFOS in Jiangsu Province *PhD Dissertation*. Peking University, 2008.

S15.	National Bureau of Statistics. China Statistical Yearbook. *Chapter 13*. Beijing: China Statistics Press; 2017.

S16.	National Bureau of Statistics. China Statistical Yearbook. *Chapter 13*. Beijing: China Statistics Press; 2018.

S17.	National Bureau of Statistics. China Statistical Yearbook. *Chapter 13*. Beijing: China Statistics Press; 2019.

S18.	National Bureau of Statistics. China Statistical Yearbook. *Chapter 13*. Beijing: China Statistics Press; 2020.

S19.	National Bureau of Statistics. China Statistical Yearbook. *Chapter 13*. Beijing: China Statistics Press; 2021.

S20.	National Bureau of Statistics. China Statistical Yearbook. *Chapter 13*. Beijing: China Statistics Press; 2022.

S21.	Wang Z, Boucher JM, Scheringer M *et al.* Toward a Comprehensive Global Emission Inventory of C(4)-C(10) Perfluoroalkanesulfonic Acids (PFSAs) and Related Precursors: Focus on the Life Cycle of C(8)-Based Products and Ongoing Industrial Transition. *Environ Sci Technol*. 2017; **51**(8): 4482-4493. doi: 10.1021/acs.est.6b06191

S22.	Ministry of Agriculture and Rural Affairs of the People’s Republic of China. Announcement No. 148 of the Ministry of Agriculture and Rural Affairs of the People’s Republic of China [in Chinese]. 2019.

S23.	Zhang L, Liu J, Hu J *et al.* The inventory of sources, environmental releases and risk assessment for perfluorooctane sulfonate in China. *Environ Pollut*. 2012; **165**: 193-198. doi: 10.1016/j.envpol.2011.09.001

S24.	Liu Z, Lu Y, Wang P *et al.* Pollution pathways and release estimation of perfluorooctane sulfonate (PFOS) and perfluorooctanoic acid (PFOA) in central and eastern China. *Sci Total Environ*. 2017; **580**: 1247-1256. doi: 10.1016/j.scitotenv.2016.12.085

S25.	Department of Rural Social and Economic Survey. Chapter 3. *China Rural Statistical Yearbook*. Beijing: China Statistics Press; 2017.

S26.	Department of Rural Social and Economic Survey. Chapter 3. *China Rural Statistical Yearbook*. Beijing: China Statistics Press; 2018.

S27.	Department of Rural Social and Economic Survey. Chapter 3. *China Rural Statistical Yearbook*. Beijing: China Statistics Press; 2019.

S28.	Department of Rural Social and Economic Survey. Chapter 3. *China Rural Statistical Yearbook*. Beijing: China Statistics Press; 2020.

S29.	Department of Rural Social and Economic Survey. Chapter 3. *China Rural Statistical Yearbook*. Beijing: China Statistics Press; 2021.

S30.	Department of Rural Social and Economic Survey. Chapter 3. *China Rural Statistical Yearbook*. Beijing: China Statistics Press; 2022.

S31.	China Land Use Remote Sensing Monitoring Data 2023. In: Resource and Environment Science and Data Center (RESDC) (ed.). Beijing: Resource and Environment Science and Data Center (RESDC),; 2023.

S32.	Buck RC, Franklin J, Berger U *et al.* Perfluoroalkyl and polyfluoroalkyl substances in the environment: terminology, classification, and origins. *Integr Environ Assess Manag*. 2011; **7**(4): 513-541. doi: 10.1002/ieam.258

S33.	Jiang W, Zhang Y, Yang L *et al.* Perfluoroalkyl acids (PFAAs) with isomer analysis in the commercial PFOS and PFOA products in China. *Chemosphere*. 2015; **127**: 180-187. doi: 10.1016/j.chemosphere.2015.01.049

S34.	Li L, Zhai Z, Liu J *et al.* Estimating industrial and domestic environmental releases of perfluorooctanoic acid and its salts in China from 2004 to 2012. *Chemosphere*. 2015; **129**: 100-109. doi: 10.1016/j.chemosphere.2014.11.049

S35.	Paustenbach DJ, Panko JM, Scott PK *et al.* A methodology for estimating human exposure to perfluorooctanoic acid (PFOA): a retrospective exposure assessment of a community (1951-2003). *J Toxicol Environ Health A*. 2007; **70**(1): 28-57. doi: 10.1080/15287390600748815

S36.	Wang Z, Cousins IT, Scheringer M *et al.* Global emission inventories for C4-C14 perfluoroalkyl carboxylic acid (PFCA) homologues from 1951 to 2030, Part I: production and emissions from quantifiable sources. *Environ Int*. 2014; **70**: 62-75. doi: 10.1016/j.envint.2014.04.013

S37. Zhang L, Zheng Z, Chen W. Feasibility Analysis and Recommendations of Disposal Technology of PFOS/PFOSF-Containing Wastes in China. *Research of Environmental Sciences*. 2022; **35**(8): 1974-1985. doi: 10.13198/j.issn.1001-6929.2022.07.08

S38.	Ministry of Ecology and Environment of China. *2020 National Ecological Environment Statistical Yearbook*. <https://www.mee.gov.cn/hjzl/sthjzk/sthjtjnb/202202/t20220218_969391.shtml> (2026-05-05)

S39.	Ministry of Ecology and Environment of China. *2021 National Ecological Environment Statistical Yearbook*. <https://www.mee.gov.cn/hjzl/sthjzk/sthjtjnb/202301/t20230118_1013682.shtml> (2026-05-05)

S40.W. Yu, P. Wang, L. Tian, X. Fu, Z. Bao, The latest information on the Stockholm Convention and an introduction to the use of PFOS in China’s fire-fighting industry [in Chinese], Fire Science and Technology 29 (2010) 513–515.

S41.Fire Department, Ministry of Public Security of the People’s Republic of China, China Fire Yearbook, 2016–2017 editions [in Chinese], Yunnan People’s Publishing House, Kunming, 2016–2017.

S42.Fire and Rescue Department, Ministry of Emergency Management of the People’s Republic of China, China Fire and Rescue Yearbook, 2018–2019 volumes [in Chinese], Emergency Management Press, Beijing, 2019–2020.

S43.Fire and Rescue Department, Ministry of Emergency Management of the People’s Republic of China, China Fire and Rescue Yearbook: 2020 Volume [in Chinese], Emergency Management Press, Beijing, 2022.

S44.Fire and Rescue Department, Ministry of Emergency Management of the People’s Republic of China, China Fire and Rescue Yearbook: 2021 Volume [in Chinese], Fangzhi Publishing House, Beijing, 2022.

S45.OpenStreetMap contributors, OpenStreetMap, OpenStreetMap Foundation, https://www.openstreetmap.org/ (accessed 15 July 2026).

S46. Secretariat of the Stockholm Convention. All POPs Listed in the Stockholm Convention. https://pops.int/TheConvention/ThePOPs/AllPOPs/tabid/2509/Default.aspx (accessed 29 July 2026).

S47. Ministry of Environmental Protection of the People’s Republic of China. Announcement on the Entry into Force of the Amendments to Annexes A, B and C Listing Nine Additional Persistent Organic Pollutants and the Amendment to Annex A Listing Endosulfan under the Stockholm Convention on Persistent Organic Pollutants [in Chinese]. 2014. https://www.mee.gov.cn/gkml/hbb/bgg/201404/t20140401_270007.htm (accessed 29 July 2026).

S48. World Bank Group. China—Reduction and Phaseout of PFOS in Priority Sectors Project: Environmental Assessment—Executive Summary of Environmental and Social Safeguard. Washington, DC: World Bank Group; 2017. https://documents.worldbank.org/en/publication/documents-reports/documentdetail/388371477681231625 (accessed 29 July 2026).

S49. Foreign Environmental Cooperation Center, Ministry of Ecology and Environment of the People’s Republic of China. Acceptance Meeting Held in Beijing for the Closed-Loop Circulation System Retrofit Demonstration Subproject in the Electroplating Sector of the China PFOS Priority Sectors Reduction and Phase-Out Project [in Chinese]. 2023. https://www.fecomee.org.cn/dtxx/xwdt/202309/t20230917_1041044.html (accessed 29 July 2026).

S50. Ministry of Environmental Protection of the People’s Republic of China. Announcement on the Release of the List of Priority Controlled Chemicals (First Batch) [in Chinese]. 2017. https://www.mee.gov.cn/gkml/hbb/bgg/201712/t20171229_428832.htm (accessed 29 July 2026).

S51. National Development and Reform Commission of the People’s Republic of China. Guidance Catalogue for Industrial Structure Adjustment (2019 Edition) [in Chinese]. 2019. https://www.ndrc.gov.cn/xxgk/zcfb/fzggwl/201911/t20191105_1327490.html (accessed 29 July 2026).

S52. Ministry of Ecology and Environment of the People’s Republic of China. Announcement on the Release of the List of Priority Controlled Chemicals (Second Batch) [in Chinese]. 2020. https://www.mee.gov.cn/xxgk2018/xxgk/xxgk01/202011/t20201102_805937.html (accessed 29 July 2026).

S53. General Office of the State Council of the People’s Republic of China. Notice of the General Office of the State Council on Issuing the Action Plan for the Control of New Pollutants [in Chinese]. 2022. https://www.mee.gov.cn/zcwj/gwywj/202205/t20220524_983032.shtml (accessed 29 July 2026).

S54. Ministry of Ecology and Environment of the People’s Republic of China. List of New Pollutants for Priority Control (2023 Version) [in Chinese]. 2022. https://www.mee.gov.cn/gzk/gz/202212/t20221230_1009192.shtml (accessed 29 July 2026).

S55. M. Li, K. Wu, J. Zhu, Z. Pang, H. Wu, G. Qiu and C. Wei. Spatiotemporal distribution, source apportionment, and driving factors of per- and polyfluoroalkyl substances in the river-to-sea systems of China Between 2004 and 2024. Water Research 290 (2026) 125032.

S56. D. Wang, X. Liu, Z. Guo, W. Shan, Z. Yang, Y. Chen, F. Ju and Y. Zhang. Legacy and Novel Per- and Polyfluoroalkyl Substances in Surface Soils across China: Source Tracking and Main Drivers for the Spatial Variation. Environmental Science & Technology 58(45) (2024) 20160-20171.

S57.	B. Tang, C. Peng, D.-J. Zhou, K.-Y. Wu, F. Tian, H.-G. Chen, S.-Y. Zhang, B.-W. Li, G.-C. Hu, M.-Z. Ren and J. Zheng. Distribution, partitioning behaviors, and source identification of legacy and emerging per- and polyfluorinated alkyl substances in the Pearl River Estuary, South China. Water Research 285 (2025) 124143.
